# Risk-Adapted Breast Screening for Women at Low Predicted Risk of Breast Cancer: An Online Discrete Choice Experiment

**DOI:** 10.1177/0272989X241254828

**Published:** 2024-06-03

**Authors:** Charlotte Kelley Jones, Suzanne Scott, Nora Pashayan, Stephen Morris, Yasmina Okan, Jo Waller

**Affiliations:** Cancer Behavioural Science Cancer Prevention Group, King’s College, London, UK; Professor of Health Psychology, Queen Mary University London, London, UK; Professor of Applied Cancer Research, Centre for Cancer Genetic Epidemiology, University of Cambridge, Cambridge, UK; Rand Professor of Health Services Research, Primary Care Unit, Department of Public Health and Primary Care, University of Cambridge, Cambridge, UK; Department of Communication, Pompeu Fabra University, Barcelona, Spain; Centre for Decision Research, Leeds University Business School, Leeds, UK; Professor of Cancer Behavioural Science, Wolfson Institute of Population Health, Queen Mary University of London, London, UK

**Keywords:** breast cancer, breast screening, conjoint analysis, discrete choice models, health care acceptability; health care preferences, health economics, medical decision making, NHS breast screening program, personal risk assessment, personalized medicine, preference elicitation methods, risk stratification, stated preferences

## Abstract

**Background:**

A risk-stratified breast screening program could offer low-risk women less screening than is currently offered by the National Health Service. The acceptability of this approach may be enhanced if it corresponds to UK women’s screening preferences and values.

**Objectives:**

To elicit and quantify preferences for low-risk screening options.

**Methods:**

Women aged 40 to 70 y with no history of breast cancer took part in an online discrete choice experiment. We generated 32 hypothetical low-risk screening programs defined by 5 attributes (start age, end age, screening interval, risk of dying from breast cancer, and risk of overdiagnosis), the levels of which were systematically varied between the programs. Respondents were presented with 8 choice sets and asked to choose between 2 screening alternatives or no screening. Preference data were analyzed using conditional logit regression models. The relative importance of attributes and the mean predicted probability of choosing each program were estimated.

**Results:**

Participants (*N* = 502) preferred all screening programs over no screening. An older starting age of screening, younger end age of screening, longer intervals between screening, and increased risk of dying had a negative impact on support for screening programs (*P* < 0.01). Although the risk of overdiagnosis was of low relative importance, a decreased risk of this harm had a small positive impact on screening choices. The mean predicted probabilities that risk-adapted screening programs would be supported relative to current guidelines were low (range, 0.18 to 0.52).

**Conclusions:**

A deintensified screening pathway for women at low risk of breast cancer, especially one that recommends a later screening start age, would run counter to women’s breast screening preferences. Further research is needed to enhance the acceptability of offering less screening to those at low risk of breast cancer.

**Highlights:**

- Risk-based breast screening may involve the deintensification of screening for women at low risk of breast cancer.
- Low-risk screening pathways run counter to women’s screening preferences and values.
- Longer screening intervals may be preferable to a later start age.
- Work is needed to enhance the acceptability of a low-risk screening pathway.

As part of a wider move toward more personalized medicine, there is increasing interest in risk-stratified screening. While current cancer screening programs offer the same screening to everyone of a particular gender and age group, risk-stratified screening would assess an individual’s risk, based on a wider range of factors, and adapt the screening offer accordingly. Women aged 50 to 70 y in England are currently offered 3-yearly breast screening, free on the National Health Service (NHS), but emerging evidence indicates that it may be safe to offer less screening for women at low risk of breast cancer.^1–5^ However, there are concerns about the potential for a negative public response to deintensified screening,^6,7^ as experienced in Australia and Wales where petitions with large numbers of signatures have been used to oppose extending cervical screening intervals from 3 to 5 y for human papillomavirus–negative women,^8^ with many women requesting screening outside the revised guidelines.^9^ Therefore, the prospective acceptability of deintensified screening for women at low risk should be comprehensively assessed.^10^

Previous research has established that women notionally support the rationale for risk-stratified breast screening (RSBS).^11–13^ However, UK and international survey studies consistently report that women find proposals to increase screening for those at higher risk more acceptable than deintensified screening for those at lower risk.^11,14–16^ Nevertheless, these studies have focused on RSBS as a whole rather than exploring the acceptability of specific risk-adapted screening pathways and how they affect particular risk groups. If risk stratification is implemented in the NHS breast screening program, policy makers will require evidence for the acceptability of this approach across the spectrum of risk.^17,18^

To date, only 1 study has exclusively focused on women’s responses to a low-risk screening pathway, which explored the acceptability of extending screening intervals (from 3 to 5 y) by interviewing participants of the BC-Predict trial^19^ in receipt of low-risk estimates (<2% 10-y risk).^20^ Although women generally accepted the rationale for less frequent screening, they highlighted the need for shared decision making and had concerns about the comprehensibility of evidence supporting the “safety” of longer screening intervals.^20^ This is consistent with qualitative findings that UK women who had not participated in breast cancer risk assessment expressed support for a low-risk screening pathway in principle. However, this was dependent on the accuracy of risk feedback and the clinical and psychological implications of being offered less breast screening.^12^ Therefore, there appears to be a degree of acceptability for a deintensified low-risk breast screening pathway but insufficient evidence on acceptability of the different ways in which the program could be modified.^21^

Previous research exploring public attitudes toward a low-risk screening pathway has generally focused on extended breast screening intervals.^20^ However, risk-based modifications that could affect other breast screening features (e.g., the age range of eligibility or imaging modality) are also under consideration.^22,23^ Investigating these different features specifically (rather than looking at deintensifying screening as a whole) and also jointly (rather than considering the features individually) will provide a more detailed evidence base for future screening policy development.

The objective of the current study was to elicit and quantify ex ante preferences for NHS breast screening programs that might be offered to the screening-eligible public at low predicted risk of breast cancer as part of an RSBS approach.^24^ The rationale for the indirect framing of the choice question, rather than an individual perspective, was to explore the societal acceptability of risk-adapted programs and mitigate possible endowment effects^25^ and/or negative emotional responses evoked by the prospect of less screening for women at low breast cancer risk.^20^ In addition to identifying and quantifying the importance of key attributes of deintensified screening, we sought to analyze the strength and direction of the effect of these attributes on preferences and predict the probability that different risk-adapted screening programs would be supported relative to current NHS breast screening recommendations. We also aimed to carry out stratified analyses to explore the relative importance of screening attributes by sociodemographic characteristics and psychosocial attitudes.^11,14,26^

## Methods

A discrete choice experiment (DCE) offers a systematic approach to elicit stated preferences by asking participants to choose between 2 or more alternatives within certain constraints (e.g., the ratio of screening benefit to harm).^27^ Each alternative is defined by a set of attributes with varying levels. The experimental design generates several hypothetical scenarios presented adjacently for respondents to choose their preferred option. As breast screening is not mandatory, the current DCE presented participants with choice sets with 2 screening alternatives for women with a low breast cancer risk and an option of no breast screening. International guidelines for the conduct of DCEs^28^ were followed throughout the development and implementation of the study. See *S7* for a DCE checklist.

### Development of the DCE Survey

#### Selection of attributes and levels

Relevant attributes were identified from prior qualitative interviews,^12^ the breast screening literature,^11,15,20,29–31^ and consultation among coauthors who are experts in epidemiological statistics (N.P.), health economics (S.M.), and risk communication (Y.O.). Five attributes were identified to include in the hypothetical low-risk programs: screening start age, screening end age, screening intervals, risk of overdiagnosis, and risk of breast cancer mortality (Table 1). Credible levels for screening start and end ages and screening intervals were guided by current RSBS trials and cohort studies (e.g., BC-Predict trial^19^ and MyPeBS^32^). Briefly, a simulated life table model of breast cancer risk^27^ was used to estimate the levels of overdiagnosis and risk of breast cancer–related mortality. In line with recommendations to enhance lay understanding of health risk statistics, small and constant denominators were used to present attribute levels.^33,34^ Full details of the identification of attributes and assignment of levels are outlined in *S1.*

**Table 1:** Attributes and Levels Used in the DCE

Attribute	Attribute Levels for the Screening Programs			Levels for No Screening
Start age for screening, y	50	55	60	No start age
End age for screening, y	65	70	—	No end age
Screening intervals	3	5	7	No interval
Risk of dying from breast cancer	24 in 1,000	25 in 1,000	26 in 1,000	27 in 1,000
Risk of overdiagnosis	2 in 1,000	6 in 1,000	10 in 1,000	0 in 1,000

Older start age, younger end age, longer screening intervals, and an increased risk of dying from breast cancer were expected to negatively affect support for risk-adapted programs. Theoretically, lower levels of overdiagnosis should increase the value of less breast screening. However, there were no a priori expectations about overdiagnosis because the concept is poorly understood by the screening-eligible public and not always considered in breast screening preferences.^35,36^

#### Experimental design and data collection

The number of potential combinations of attributes with one 2-level attribute, three 3-level attributes, and one 4-level attribute is 216 (2^1^ × 3^3^ × 4^1^). Two options to choose from in each choice question gives a possible 46,440 choices (216 × 215). To reduce choices to a manageable number, a fractional design was applied using the –dcreate– Stata command,^37^ which creates efficient DCE designs. This reduced the design to 16 pairs of programs, which were split into 2 blocks of 8 with half the participants randomly assigned to each block. The experimental design is illustrated in *S2.*

A marketing and research agency specializing in tradeoff analysis, Accent Marketing & Research Ltd. (https://www.accent-mr.com/), programmed and hosted the DCE on their Web-based platform.

#### Sample population and recruitment

Participants were eligible if they were women aged between 40 and 70 y with, or soon to have, the experience of deciding whether or not to attend NHS breast screening. Participants were also required to be UK residents and able to consent and have internet/smartphone access (to complete the survey). Those with a current or prior diagnosis of breast cancer and/or first-degree family history of breast cancer were excluded as these groups are unlikely to be low risk with RSBS.

Although there is no definitive calculation to establish a DCE sample size,^38^ the Orme approximate formula^39^ was used to estimate a minimum sample size for a fixed-effects model (*N* = 83). To allow for stratified analyses by sociodemographic, psychosocial, and experiential characteristics, the target sample size was increased to *N* = 500 in line with previous DCEs exploring breast screening preferences^29–31^ and available resources. An online panel, Savanta (https://savanta.com), was used to recruit study participants. As it was important that the risk-adapted screening programs were considered by women with a range of breast screening experience and socioeconomic backgrounds, sampling quotas were specified for age group as a marker of breast screening eligibility (33.3% per decade between 40 and 70 y of age) and National Readership Survey occupational social grades^40^ dichotomized to ABC1 and C2DE, representing higher and lower social grades, respectively.

To mitigate satisficing and “speeders,” minimum times were set for Web pages containing study information and the practice choice tasks, so participants were unable to skip these sections by going straight to the main choice tasks, and a minimum survey completion time of 8 min was imposed. No time limits were attached to the choice tasks. The IP address of each eligible participant was collected to avoid serial responders. All participants were offered links to further information and emotional support and reimbursed (∼£1) for their time.

#### Study procedure and other measures

We collected demographic information, including current age, occupational social grade,^40^ highest educational attainment, ethnic background, and relationship status, as these factors are associated with attitudes toward breast cancer and screening.^41–43^ As illness representations and awareness of breast cancer screening may influence screening preferences,^11,13,15^ perceived personal breast cancer risk, breast cancer worry, and prior awareness of overdiagnosis were assessed. Participants were also asked to disclose breast screening behavior and personal experience of close relatives/friends with breast cancer.

**Figure 1:**
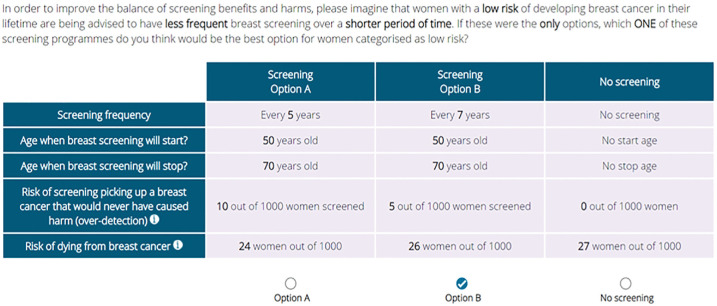
An example of a choice set as presented to online participants.

Before the DCE choice tasks, participants viewed the following background information and study training materials: 1) “What is the NHS Breast Screening Programme, and how does it work?” described the main benefits and harms of breast screening; 2) “How can the NHS Breast Screening Programme be improved?” presented an infographic to demonstrate how personal risk information may improve the precision of breast screening; 3) “How would this personal risk information be used?” and 4) “What would a risk-based screening programme look like?” illustrated how breast screening recommendations may be tailored to levels of personal risk with a “risk ladder,” allowing participants to contextualize low-risk screening recommendations vis-à-vis those for higher risk groups.^44,45^ The training materials informed participants that the study focused on breast screening strategies for women assessed to have a low lifetime risk as part of a hypothetical RSBS program. The choice question was framed from a societal perspective^24^: “If these were the **only** options, which **ONE** of these screening programmes do you think would be the best option for women categorised as low risk?” (Figure 1). All participants were asked to complete a practice question before proceeding to the 8 main choice sets.

Following the main choice tasks, participants completed 2 measures rating their experience of the DCE survey in terms of overall task complexity and how easy or difficult they found deciding between the screening programs.^29^ Finally, participants were asked to rate their subjective understanding of the risk of overdiagnosis and whether they had been aware of this screening-related harm prior to study participation. The survey closed with a brief outline of the study aims, reiterating the hypothetical nature of the programs and assuring participants that there are no imminent changes to the NHS Breast Screening Programme. The DCE survey template and a list of non-DCE measures are available on the Open Science Framework (https://osf.io/kcv9g/) and in *S3.*

The survey was pilot tested with a convenience sample of 40 participants.

Descriptive analysis of the pilot data demonstrated participant usability and coherence. Conditional logit regression modeling revealed that the preference weights were coherent and in the expected direction. Consequently, no further adjustments were required, and the main DCE survey was conducted in February 2022. Responses from the pilot study were not included in the full sample for final analysis.

The study was granted ethical approval by the Bio-medical Panel, King’s College London (Ref: LRS-21/22-25904).

### Statistical Analyses

Descriptive statistics for the characteristics of participants who completed the questionnaire were computed.

The attributes of start age, end age, intervals, and risk of overdiagnosis showed a nonlinear distribution and were modeled as categorical variables. By contrast, risk of dying from breast cancer was analyzed as a continuous variable. As the outcomes of the mixed logit models (*S4*) revealed no random effects (likelihood ratio tests *P* > 0.05), a fixed-effect conditional logit regression model^46^ was conducted to analyze the preference data, whereby the outcome variable was screening preference (options A or B, or no screening). The “no screening” option was included as the alternative specific constant term to reflect the average effect of the unobserved screening attributes on choosing not to participate in screening relative to the 2 screening alternatives.

The relative importance of risk of dying was calculated by subtracting the product of the beta coefficient for the lowest risk level (24/1,000) from that for the highest level (27/1,000). For start age, end age, intervals, and risk of overdiagnosis, the relative attribute importance (RAI) was defined by the range in beta coefficients between the highest and lowest levels.

Simultaneous conditional logit regression models were conducted for exploratory stratified analysis by sociodemographic, psychosocial, and survey-related characteristics. Between-group differences in the regression coefficients were analyzed with χ^2^ tests. To control for type 1 error, the level of statistical significance for the χ^2^ tests was adjusted to *P* < 0.001. RAI calculations were used to standardize the regression coefficients, although we did not formally analyze between-group differences in RAI.

Regression coefficients were also used to calculate the predicted probabilities that the 32 low-risk screening programs would be chosen over current NHS breast screening. Attributes for the latter were defined as start age 50 y, end age 70 y, 3-y screening intervals, risk of overdiagnosis 10/1,000, and risk of dying from breast cancer 24/1,000 out of women screened. The screening programs were ranked in order of the predicted probability they would be supported relative to current NHS breast screening recommendations.

Descriptive statistics were analyzed using IBM SPSS-26. The predicted probabilities were calculated using Microsoft Office365 Excel. All other analyses used Stata version 18.0 (StataCorp, College Station, TX, USA).

## Results

### Participant Characteristics

A total of 502 women aged between 40 and 70 y completed the survey. Participants had a mean age of 55 y (standard deviation [*s*] ± 9.37) and varied levels of educational attainment. Most were married/in a civil partnership and from a white ethnic background (Table 2).

**Table 2:** Participant (*N* = 502) Sociodemographic, Breast Cancer–Related, and Survey-Related Characteristics

Characteristic	*n* (%)
Socio-demographic characteristics	
Age, y	
40–50	166 (33.1)
51–60	170 (33.9)
61–70	166 (33.1)
Exact age, y, $\bar x$±*s*	55.0 ± 9.4
Occupational social grade	
Higher social grade (ABC1)	252 (51.0)
Lower social grade (C2DE)^a^	246 (49.0)
Educational attainment	
No formal qualifications	32 (6.4)
O’ levels/GCSE^b^	171 (34.1)
A’ levels^b^	87 (17.3)
Higher education below degree	88 (17.5)
Bachelor’s degree	93 (18.5)
Further degree	24 (4.8)
Still studying	1 (0.2)
Other	6 (1.2)
Relationship status	
Single	72 (14.3)
Married/civil partnership/living with partner	327 (65.1)
Divorced/separated/widowed	102 (20.3)
Other	1 (0.2)
Ethnicity	
White ethnic background	481 (95.8)
Mixed/multiple ethnic backgrounds	5 (1.0)
Asian background	9 (1.8)
Black background	6 (1.2)
Arab/other ethnic group	1 (0.2)
Breast screening and breast cancer–related participant characteristics	
Breast screening experience	
Not yet eligible	160 (31.9)
Never attended	27 (5.3)
Occasional attender	34 (6.8)
Regular attender	281 (56.0)
Perceived risk	
Lower than average	56 (11.2)
Average	315 (62.8)
Higher than average	56 (11.2)
Don’t know	75 (14.9)
Breast cancer worry	
Low	79 (15.7)
Moderate	283 (56.4)
High	140 (27.9)
Breast cancer experience^c^	
One experience of breast cancer	198 (39.4)
Multiple experiences of breast cancer	227 (45.2)
No experience of breast cancer	138 (27.5)
Prior awareness of overdiagnosis	
Yes	199 (39.6)
No	257 (51.2)
Not sure	46 (9.2)
Survey experience	
Subjective understanding of survey task	
Easy/very easy	342 (68.1)
Fair	95 (18.9)
Difficult/very difficult	65 (12.9)
Choice task experience	
Easy	213 (42.4)
Fair	99 (19.7)
Difficult	190 (37.9)
Subjective understanding of information about overdiagnosis	
Easy	439 (87.5)
Hard	63 (12.5)
Mean time spent completing survey, min, $\bar x$±*s*	11.2 ± 5.4 (range, 07.0–48.4)

a Occupational social grade as specified by the National Readership Survey system.^40^

b O’ levels/GCSEs represents 12 y of compulsory schooling in England, after which individuals may choose to continue schooling for 2 y to gain A’ levels.

c Breast cancer experience refers to experiencing family members, friends, and close colleagues with a diagnosis of BC.

Most participants were either not yet eligible for breast screening (*n* = 160, 32%) or regular screening attendees (*n* = 281, 56%), with about 12% (*n* = 61) representing occasional or nonattenders. Although more than half of the participants considered their breast cancer risk to be “average” (*n* = 315, 62.7%), about 11% (*n* = 56) reported higher or lower perceived risk in equal measures. More than 80% reported high (*n* = 140, 28%) or moderate (*n* = 283, 56%) levels of breast cancer worry. About a third (*n* = 138, 38%) had no personal experience of friends or family with a breast cancer diagnosis. Just more than half the participants were unaware of the risk of overdiagnosis before study participation.

### DCE Experience and Data Quality

About two-thirds (*n* = 342, 68%) of participants found the DCE tasks easy to understand, with only 13% (*n* = 65) reporting they found completing the choice questions difficult. By contrast, less than half the sample (*n* = 213, 42%) found deciding between the 3 screening options straightforward.

Nineteen participants (4%) made the same choice (i.e., screening option A or B) for all 8 choice sets. As this is a relatively small number and the possibility exists that these responses could be logical preferences, they were not excluded from the analyses.

To check the impact of “speeders,” a sensitivity analysis was conducted by excluding 24% (*n* = 121) of participants who completed the survey in under the minimum completion time of 8 min (see *S4*). Excluding these responses had no significant impact on the strength or direction of preference weights, so all participants (*N* = 502) were included in the final analyses.

### Attribute Valuations and Preferences

All participants completed 8 choice sets providing 4,016 (502 × 8) choices and 12,048 observations, that is 4,016 × 3 (A, B, and no screening). As hypothesized, regression coefficients representing attribute preferences demonstrated that, compared with current age of screening eligibility (50 to 70 y), an older start age (55 and 60 y) and younger end age (65 y), extended screening intervals (7 rather than 3 y), and increased risk of dying from breast cancer had a negative impact on preferences (all *P* values < 0.01; Table 3). By contrast, a 4-point decrease in the risk of overdiagnosis (6/1,000 v. 10/1,000) increased the value of screening programs for low-risk groups (*P* = 0.04).

**Table 3:** Coefficients and Direction of Preferences by Breast Screening Attribute: Number of Observations 12,048 (502 Clusters)

Attribute		Level	Coefficient	*P* Value^a^	95% CI	
Start age for screening		Age, y				
50	*ref*					
55			−0.14	0.003	−0.23	−0.05
60			−0.67	0.001	−0.77	−0.56
End age for screening		Age, y				
70	*ref*					
65			−0.34	0.001	−0.41	−0.27
Screening intervals		Years				
3	*ref*					
5			0.01	0.92	−0.08	0.09
7			−0.47	<0.01	−0.59	−0.36
Risk of dying from breast cancer		Absolute risk	−0.16	<0.01	−0.20	−0.12
Risk of overdiagnosis						
10 in 1,000	*ref*					
6 in 1,000			0.10	0.04	0.01	0.19
2 in 1,000			<0.01	0.99	−0.09	0.10
Alternative specific constant			−2.28	<0.01	−2.46	−2.11

a The likelihood ratio χ^2^ test, χ^2^(9) = 2,060.91, *P* < 0.001, indicated that the inclusion of attribute levels significantly improved the model. The pseudo *R* ^2^ of 0.23 indicates a good relative model fit.

Neither the extension of screening intervals from 3 to 5 y nor the lowest level for the risk of overdiagnosis (2/1,000) influenced screening preferences (*P* > 0.05). The negative direction and statistical significance of the alternative specific constant term indicates participants’ propensity to choose the screening alternatives A or B over no screening (*P* < 0.001), which was chosen in only 6.1% of cases (243/4,016).

### RAI

The calculation of the RAI (Table 4) demonstrated that an earlier start age was the most valued feature of the screening programs (0.67), followed by risk of dying from breast cancer (0.48), shorter screening intervals (0.44), and later end age (0.34). The influence of risk of overdiagnosis on preferences was minimal (0.10).

**Table 4:** Heat Map Showing the Relative Attribute Importance (RAI) of Screening Programs for All Participants (*N* = 502) and for Subgroups in Which There Were Significant Between-Group Differences (χ^2^, *P* < 0.001)^a^

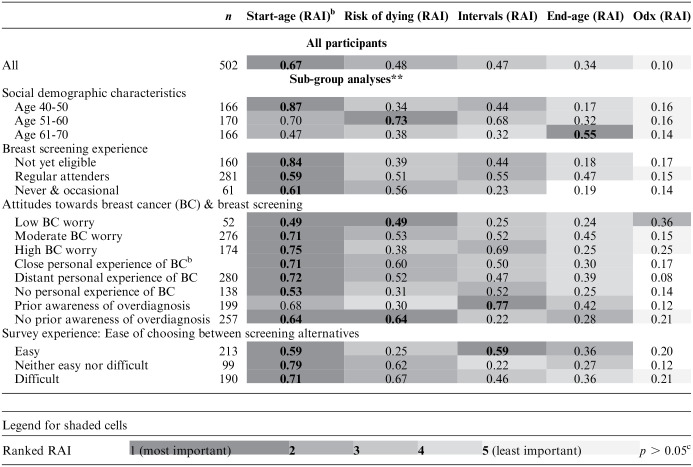

a Please note comparisons for the numbers of participants in each stratified group (*n*) can only be made row-wise.

b Distant personal experience includes distant family relatives, acquaintances and colleagues rather than close family members, partners or close friends.

c No significant impact of attribute coefficient when participants were stratified by group characteristics (*p* > 0.05).

### Regression Coefficients and Relative Attribute Importance by Subgroup

Simultaneous conditional logit regression analyses found significant differences in preference coefficients (χ^2^, *P* < 0.001) when participants were stratified by age group, breast screening experience, and attitudes toward breast cancer (breast cancer worry, personal experience of friends and family with breast cancer, and prior awareness of overdiagnosis). There were also significant group differences by how easy participants found choosing between screening programs. Differences in RAI with participants stratified by group characteristics are illustrated by way of a heat map (Table 4).

RAI calculations for the stratified groups showed a trend for most subgroups valuing screening start age and risk of dying from breast cancer as the most important attributes. However, there were notable exceptions. For example, among those aged 61 to 70 y, end age of screening was the most influential attribute, while for those with low levels of breast cancer worry, the risk of overdiagnosis had a greater influence than screening intervals and screening end age. The length of screening intervals was considered to be the most important feature by participants with prior awareness of overdiagnosis and those who found the choice task easy. For both of these groups, extending screening intervals from 3 to 5 y had a negative impact on preferences (*P* < 0.05).

There were also trends for differences in the relative importance of risk of dying and screening intervals, with younger age groups not yet eligible for NHS breast screening and those with higher levels of worry valuing shorter screening intervals over risk of dying.

There was variation in the strength and directionality of the influence of overdiagnosis on preferences. For instance, a lower risk of overdiagnosis increased support for risk-adapted screening programs among participants with low breast cancer worry (*P* < 0.001) and those who found choosing between the screening alternatives “difficult” (*P* < 0.01). Conversely, and unexpectedly, a lower risk of overdiagnosis had a negative impact on support for a screening program among those with high levels of breast cancer worry (*P* = 0.01). See *S5* for full details of subgroup analyses.

### Mean Predicted Preference Probabilities

The mean predicted probabilities of participants supporting the risk-adapted programs relative to current NHS screening recommendations are illustrated in Table 5, which includes 10 screening programs considered to be clinically plausible in terms of future breast screening policy and are ranked in order of preference (see *S6* for all 32 screening programs).

**Table 5:** Mean Predicted Probabilities of Participants Supporting 10 Risk-Based Breast Screening Programs Relative to the Current NHS Breast Screening Programme^a^

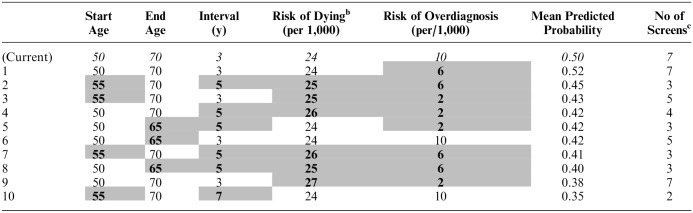

a Risk-adapted changes from current attribute levels (italicised text) are indicated by gray-shaded cells and bold text.

b From breast cancer ^c^excluding initial mammography as part of personal risk assessment.

Overall, participants were more likely to support programs closest to current NHS screening. Therefore, the predicted mean probabilities for all deintensified screening programs were low (range 0.52 to 0.18). Program 1 was most likely to be supported (predicted probability 0.52) as this has marginally more favorable attribute levels than those of the NHS program (i.e., a 4 -unit decrease in risk of overdiagnosis with all other attributes being the same; Table 5).

There was moderate support for starting screening at 55 y rather than 50 y (programs 2 and 3), as long as risk of dying, screening intervals, end age, and risk of overdiagnosis were not at their highest levels. Likewise, an earlier end age of 65 y and 5-y screening intervals (programs 3 and 4) may be supported if the increased risk of dying is minimal. It is notable that participants would be less likely to support program 8, which offers the same amount of screening as currently recommended but with the highest risk of dying (27/1,000) than programs offering fewer screens. Nevertheless, low probabilities for supporting 7-y screening intervals indicate that the number of screens is important in itself. For example, the probability of supporting program 10 with 3 screens is low (0.35) despite having the lowest risk of dying from breast cancer (24/1,000).

The predicted probabilities of participants supporting programs with a screening start age of 60 y were low irrespective of risk of dying and overdiagnosis.

## Discussion

The results of this DCE demonstrated that, regardless of information outlining the equivocal balance of benefit-to-harm of screening for women identified to be at low risk of breast cancer, an overwhelming majority of participants considered the risk-adapted screening programs to be a better option than no screening. Preference coefficients for the modeled attributes demonstrated that an older screening start and younger end age, extension of screening intervals from 3 to 7 y, and increased risk of breast cancer mortality adversely affected the likelihood of a program being supported. This was further reflected by the low mean predicted probabilities that women would support screening programs for low-risk groups compared with the option mirroring current NHS breast screening. Despite subgroup analysis demonstrating differences in the strength of attribute coefficients, the negative direction of screening coefficients was largely constant when participants were stratified by sociodemographic characteristics, psychosocial attitudes, and survey-related experience.

There was high subjective understanding of the information provided on overdiagnosis, and about 40% claimed to have prior awareness of this screening-related harm. However, a conditional logit model including all participants demonstrated that its relative importance was minimal. Moreover, subgroup analysis showed that those with prior awareness of overdiagnosis valued reduced screening intervals over other attributes, which, given the rationale for offering less screening for low-risk groups, is somewhat paradoxical. Nevertheless, in some cases exploratory subgroup analyses indicated that a lower risk of overdiagnosis would increase the value of screening alternatives, especially among participants with low levels of breast cancer worry. The risk of overdiagnosis may also have informed the screening preferences of women with no prior awareness of this screening harm and those who found the process of deciding between the screening alternatives challenging. However, subgroup analysis revealed that the direction and significance levels of coefficients for the risk of overdiagnosis was mixed.

Collectively, these results are consistent with findings that this harm is poorly understood and not always considered in breast screening preferences.^35,36^

The observed trends for group differences in RAI corresponds with previous DCEs eliciting preferences for current screening recommendations, which vary by sociodemographic characteristics and psychosocial attitudes toward breast cancer and screening.^29–31,47,48^ The extension of such differences in screening preferences to deintensified screening for women at low risk suggests that future strategies to evaluate cognitive and emotional responses to less screening may need tailoring to preference-based subgroups.

Although not directly comparable, the current DCE results reflect low levels of acceptability for deintensified breast screening relative to UK and international attitudinal survey studies, which have explored public responses to RSBS as a whole.^11,13–16,49,50^ Nevertheless, these studies have largely asked women to indicate their support for extended screening intervals only. By contrast, this DCE presented participants with multiple variations of 5 screening attributes and their levels, which required compensatory decision making. While this study design aimed to offer more contextual detail, it is possible that the presentation of specific screening programs increased aversion toward the prospect of deintensified screening by making this more “real.”

A recent survey of women with low-risk estimates as part of the UK’s Predicting Risk Of Cancer At Screening study (PROCAS)^19^ found that 56% would prefer to maintain the current screening interval of 3 y rather than have screening less frequently with a reduced risk of screening harm.^50^ Furthermore, the low mean predicted probability for women supporting a risk-adapted screening program with more favorable attribute levels—a 4-unit decrease in risk of overdiagnosis with all other attributes being the same—than those currently offered by NHS breast screening implies that the inclusion of levels for younger start age, shorter screening intervals, and older end age would likely have yielded higher estimates of probability. These findings correspond to a “more the better” approach to breast screening associated with high levels of tolerance for screening-related harms^51–54^ and is consistent with previous DCEs investigating women’s preferences for age-based breast screening. For instance, a DCE indicated that women in France would tolerate 14 additional cases of overdiagnosis and 48 false-positive recalls to avert 1 breast cancer–related death.^30^ Likewise, a UK DCE found that two-thirds of women preferred a worst-case screening scenario in which the risk of unnecessary follow-up was 20% higher than a true-positive result. Moreover, 4% of that sample indicated that they would prefer screening even with a “catastrophic” scenario of 0% benefit of screen-detected disease and a 100% chance of unnecessary follow-up.^29^ These findings are in line with the so-called “action bias,” a psychological phenomenon whereby people prefer action over inaction, potentially leading to a preference for screening over no screening, even when screening does not save lives and only causes harms.

The current findings also suggest that information alone may not mitigate women’s relative aversion to a low-risk breast screening pathway. Preconceptions about breast cancer can influence the impact of information about breast screening harms, especially if this conflicts with previously held beliefs and social norms^55,56^ (e.g., breast cancers are always fatal and breast screening is the only way to avoid dying from breast cancer. As the present study did not control for the influence of information on screening preferences, this may be an opportunity for further research.

DCEs assume that individuals make rational decisions to maximize gain rather than intuitively on an affective and/or experiential basis.^57–59^ However, research has shown that affect plays a key role in decision making, often shaping judgments of risks and benefits of interventions such as cancer screening.^60,61^ Although subgroup analyses suggest emotional factors (e.g., fear) may have had an impact on screening preferences, these may not have fully captured the influence of affective attitudes on preference outcomes. Moreover, the fear associated with cancer risk may, in some cases, lead people to neglect the probabilistic information provided.^62^ Therefore, future efforts to elicit preferences for deintensified screening programs should be sensitive to women’s health values and emotional concerns about breast cancer.^63^ This may require innovative research approaches,^64^ for instance, DCEs based on alternative decision rules such as regret minimization approaches.^65,66^ Other approaches include the incorporation of a DCE in a decision aid to “nudge” women toward value-concordant breast screening choices.^67^ Preference elicitation methods could also be optimized by including more participatory elements (e.g., citizens’ juries and deliberative polls).^68,69^

### Strengths and Limitations

The hypothetical nature of DCEs to elicit health care preferences is both a strength and a limitation. Without revealed preference data, DCEs can provide a less time-consuming and more cost-effective assessment of choices than pilot studies.^70^ For this reason, such methods are criticized as hypothetically biased and lacking external validity.^70,71^ Nevertheless, we attempted to minimize hypothetical bias by including participants representative of real-world decision makers and realistic ratios of benefit-to-harm to reflect the tradeoffs women at low risk may need to make.

As it was unfeasible to include attributes valued by every participant,^72,73^ it is possible that further attributes (e.g., the number of lives saved from breast screening or frequency of false-positive results) may have better reflected women’s screening values. Furthermore, the reported preference coefficients and RAI values are specific to the context of this study design. In this respect, the current findings should be regarded as indicative, with further research required to assess external validity.

Although variation in preferences by participant characteristics was anticipated, the current DCE may not have been sufficiently powered for such extensive subgroup analyses. Moreover, we did not control for confounding effects (e.g., the interaction between age and breast screening experience). Therefore, subgroup analyses are exploratory with further research, such as latent class analysis, required to fully investigate the acceptability of deintensified screening by sociodemographic and psychosocial characteristics.

Importantly, study participants were asked to decide between risk-adapted screening programs from a societal perspective, which may not have been concordant with preferences for their own screening.^24,74^ When considering others, decision making could be more risk averse to avoid deterring others from breast screening.^74–76^ Consequently, further research is required to investigate the potential for self-other decisional asymmetry.

Although the choice tasks were informed by evidence-based recommendations to support understanding, the numerical nature of the attribute levels may have been cognitively overwhelming for some.^77,78^ Due to time and resource constraints, we did not conduct cognitive interviews or focus groups as an adjunct to the DCE survey, which may have offered valuable insight into participants’ decision making.^63,78–80^

Finally, online panels have been criticized for compromising the quality of research data.^81^ However, the relatively small number of “satisficers” and “speeders”^73^ suggests that the quality of our choice data is reasonable. Nonetheless, an overwhelming majority of participants were from white ethnic backgrounds, which limits the generalizability of the findings.

## Conclusions

Women’s preferences and values should be taken into account when considering changes to national breast screening programs to maximize acceptability in line with the UK National Screening Committee’s criteria. Based on the results of the current study, a deintensified screening pathway for women at low risk of breast cancer, especially one that recommends a later screening start age, would run counter to women’s preferences. As the cognitive emphasis of a DCE design may not have captured the complex nature of women’s screening decision making, it is evident there is a need to research ways to enhance the acceptability of risk-adapted changes to the NHS Breast Screening Programme that propose offering less breast screening to those with a low risk of breast cancer.

## Supplemental Material

sj-docx-1-mdm-10.1177_0272989X241254828 – Supplemental material for Risk-Adapted Breast Screening for Women at Low Predicted Risk of Breast Cancer: An Online Discrete Choice Experiment Supplemental material, sj-docx-1-mdm-10.1177_0272989X241254828 for Risk-Adapted Breast Screening for Women at Low Predicted Risk of Breast Cancer: An Online Discrete Choice Experiment by Charlotte Kelley Jones, Suzanne Scott, Nora Pashayan, Stephen Morris, Yasmina Okan and Jo Waller in Medical Decision Making
