# Supplementary material for: Risk-Adapted Breast Screening for Women at Low Predicted Risk of Breast Cancer: An Online Discrete Choice Experiment
Source: Med Decis Making. 2024 Jun 3;44(5):586–600. doi: 10.1177/0272989X241254828 (PMC11283735; doi:10.1177/0272989X241254828)
Supplement: sj-docx-1-mdm-10.1177_0272989X241254828 – Supplemental material for Risk-Adapted Breast Screening for Women at Low Predicted Risk of Breast Cancer: An Online Discrete Choice Experiment [file sj-docx-1-mdm-10.1177_0272989X241254828.docx]

# Supplementary materials

## **S1. Identification of attributes and assignment of attribute levels**

Five attributes, screening start age; screening end age, screening intervals, risk of breast cancer mortality, and risk of overdiagnosis, were included in the DCE and categorised as either screening programme or test outcome attributes.

### **Breast Screening programme attributes**

As evidence has yet to establish the extent to which the natural history of breast cancers varies by adapting breast screening intervals to a personal level of risk (Noguchi et al., 2022), and how the trade-offs for extending screening intervals may vary by age group (Pashayan et al., 2021a; Schünemann et al., 2020), it could be useful for future screening policy-makers to learn how women value potential changes to both these features of breast screening.

Age-range of screening eligibility: start age and end age.

It is possible that women at lower risk may clinically benefit from undergoing breast screening over a shorter period of time (Canelo-Aybar et al., 2022; Pashayan et al., 2021a). However, the interview study suggested that women have mixed attitudes towards shortening the age-range of screening eligibility, with more women preferring an earlier end age to breast screening over a later start age. It was also evident that interviewees had greater awareness of the current starting-age of 50 relative to the age of ending screening at 70, which is consistent with prior research assessing women’s knowledge about breast screening (Seaman et al., 2018). Notwithstanding, several women expressed concern at the prospect of no longer receiving invitations for breast screening after 70 years of age. These concerns appeared to be driven by a limited understanding about the risk of breast screening and feelings of being abandoned by the NHSBSP (Kelley‐Jones et al., 2021). Therefore, it was important to investigate how the impact of both the start age and end age of screening eligibility informed women’s preferences for low-risk screening options and how these attributes interacted with other risk-stratified screening features.

*Screening intervals*

Although previous research and our interview study have found that women appear to pay more attention to risk-adapted screening intervals than the age-range of screening eligibility (Kelley‐Jones et al., 2021; Rainey et al., 2020), few studies have addressed how these two screening attributes may interact to inform women’s screening preferences. This is also important as the introduction of risk-stratified breast screening might involve key changes to both age-range and screening intervals (Pashayan et al., 2021a).

### **Breast screening outcome attributes**

*Risk of dying from breast cancer*

A reduced risk of breast cancer death is the key benefit of screening. The tendency of women to overestimate both their risk of breast cancer (Davidson et al., 2011; MASCara & Constantinou, 2021; McMenamin et al., 2005; Silverman et al., 2001), and the impact of breast screening on breast cancer mortality has been well documented (Gigerenzer et al., 2009; McMenamin et al., 2005; Rozbroj et al., 2020, 2021; Seaman et al., 2018). Therefore, the inclusion of credible levels of breast cancer mortality may be key to women’s cognitive assessment of other low-risk screening attributes.

*Risk of overdiagnosis*

Low levels of awareness and understanding about overdiagnosis are also well-documented (Jansen & Houssami, 2018; Mathioudakis et al., 2019; Rozbroj et al., 2021; Seaman et al., 2018). Although the potential of RSBS to reduce levels of over-diagnosis is fundamental to its rationale, few studies assessing women’s attitudes towards this screening approach have outlined this screening-related harm (Ghanouni et al., 2019; Kelley‐Jones et al., 2021; Wegwarth et al., 2018).

### **Levels for breast screening programme attributes**

*Age range of screening eligibility and screening intervals*

Research is currently exploring how age-based risk thresholds could be instrumental in translating the results of PRA into screening strategies (Pashayan et al., 2021a). Therefore, there may be scenarios whereby women at the same level of risk are offered different breast screening recommendations.

The periodicity of screening eligibility may be one of the features of breast screening that could vary for women with a low breast cancer risk (Canelo-Aybar et al., 2022; Klarenbach et al., 2018; Pashayan et al., 2021b; Van Den Broek et al., 2021; Zielonke et al., 2021). As many participants in the interview study were not always aware of the current age range of eligibility of NHS breast screening, and research has yet to quantify women’s preferences for this, a screening start age of 50 and end age of 70 were included as levels in the DCE model. As PRA estimated for a fixed time (e.g., 5 or 10 year absolute risk), hypothetical levels of 55 and 60 were set for screening start age and an earlier end age of 65 were set for inclusion in the DCE.

Ongoing prospective cohort studies and trials are testing extended intervals ranging from 4 to 5 years. In the UK, BC-Predict (French et al., 2020) is currently exploring RSBS responses to 5-year screening intervals for women with a low predicted risk. This has been determined, in part, for pragmatic reasons, to allow women at low-risk to be invited for breast cancer screening in alternate rounds (McWilliams et al., 2021; Woof et al., 2021). MyPeBS (Rouge-Bugat et al., 2022; UNICANCER, 2017) proposes less than one mammography screening over a period of 4-years for women with low-risk feedback which is an increase of two years from the current age-based screening European guidelines. However, this would only represent extending the current NHS screening interval by a year for UK women with low-risk feedback. As NHS screening intervals for low-risk groups are likely to be longer than a year, it was decided to include an extended interval of 5 years in the DCE design. As no screening is also being considered as an option for women at very low risk (Evans et al., 2012; Pashayan et al., 2018), a longer interval of 7 years was also included to explore women’s responses to this in relation to a decreased risk of overdiagnosis and a marginal increase in the risk of dying from breast cancer.

### **Levels for breast screening outcome attributes**

*Risk of dying from breast cancer and risk of overdiagnosis*

Evidence from empirical data is still required to determine the absolute risk thresholds that may be used for RSBS programmes. Therefore, the benefits and harms for this experiment were estimated on the basis of a simulated life-table model of breast cancer risk (Pashayan et al., 2018), and incorporated three potential thresholds for defining low absolute lifetime risk for developing breast cancer; <3.6%, <6% and <6.5%, which represent 4%, 20% and 25% of the UK screening eligible population, respectively. As a low-risk threshold of <3.6% would have minimum effect, because only 4% of women would be classed as low-risk, this serves as a base-level and is aligned with screening outcomes for age-based screening which are estimated to equate to 238 breast cancer deaths and 104 cases of overdiagnosis per 10,000 women screened between the ages of 50 and 69 (Pashayan et al., 2018).

Modelling suggests that a risk-stratified approach has the potential to reduce the risk of overdiagnosis to 89 (-15) and 84 (-20) for risk thresholds of <6% and <6.5%, respectively. However, this would entail marginal increases to risk of death from breast cancer which would be 238(for <3.6%); 241 (for <6%) and 242 (for <6.5%) per 10,000 women screened. These figures are based on a model of risk-stratified breast with a common screening strategy of triennial screening for all levels of risk between the age of 50 and 70 years which, in turn, are extrapolated from the UK Independent Panel on breast cancer screening (Marmot et al., 2013). Nonetheless, they provided: “*a reference to accordingly use hypothetical numbers for* [the risk of] *overdiagnosis and breast cancer deaths for the other levels*” (Pashayan, 2021, personal communication).

As lay understanding of health risk statistics have established that the use of large denominators (e.g., 10,000) may be cognitively overwhelming for survey respondents (Trevena et al., 2021; Witteman et al., 2021), the estimated levels for risk of dying from breast cancer and overdiagnosis were adapted to a denominator of 1000 women. In order to account for the possibility of greater variation in the risk of dying from breast cancer with risk-based modifications to screening intervals and age range of screening eligibility, alongside the need to include sufficient strength of stimuli, the increased risk of dying were set at 24, 25, 26 and 27 per 1,000 low-risk women. Although these were slightly larger estimates than those modelled, they still within the range of difference (i.e., 1-3) between ABS and RSBS as modelled by Pashayan et al (see *Table S1.1.)* As the benefit of screening for reduced BC mortality may be minimal for women with a low risk, the highest level of risk of dying (27/1000) was also set for the ‘no screening’ alternative. Furthermore, it was anticipated that this would assist the assessment of whether women would prefer screening even where the risk of dying from breast cancer was not reduced.

The modelled estimates for the reduced risk of overdiagnosis were caveated by the lack of evidence between low-risk status and the progressiveness of breast cancers, consequently a conservative range of difference was adopted for this attribute between the two screening alternatives with the setting of levels at 2, 6 and 10 (see *Table S1.2)*.

*Table S1.1*. Determination of hypothetical levels of risk of breast cancer mortality and risk of overdiagnosis using modelled estimates (in bold) as reference points.

|  | Denominator | ABS  Frequencies *[diff]* (%) | Low risk <3.6%  Frequencies *[diff]* (%) | Low risk <6%  Frequencies *[diff]* (%) | Low-risk <6.5%  Frequencies *[diff]* (%) |
| --- | --- | --- | --- | --- | --- |
| Risk of BC death | **10,000** | **238** (2.38) | **238** (2.38) | **241** *[+3]* (2.40) | **242** *[+4]* (2.4) |
|  | 1,000 | 24 (2.4) | 25 [+1](2.5) | 26 *[+2]* (2.6) | 27 *[+3]* (2.7) |
| Risk of overdiagnosis | **10,000** | **104** (1.4) | **102** [-2] (1.2) | **89** [-15] (0.89) | **84** [-20] (0.84) |
|  | 1,000 | 10 (1.0) | 10 (1.0) | 6 *[-4]* (0.6) | 2 *[-4]* (0.2) |

*Table S1.2*. DCE attributes, regression coding, levels, and modelling

| *Attributes* | *Regression label* | *Levels* | *Variable coding* |
| --- | --- | --- | --- |
| **Breast screening programme** | | | |
| Start age for screening | start | 50, 55 & 60 years | Categorical (dummy-variable coding) |
| End age for screening | end | 65 & 70 years | Categorial  (dummy-variable coding) |
| Screening intervals | interval | 3, 5, & 7 years | Categorical  (dummy-variable coding) |
| **Breast screening test outcomes** | | | |
| Risk of dying of breast cancer | riskdie | 24, 25, 26, & 27 in 1,000 | Naturally ordered |
| Risk of over-diagnosis (treatment for a cancer that never would have caused harm) | odx | 2, 6, & 10 in 1,000 | Naturally ordered |

***References***

Bridges, J. F. P., Hauber, A. B., Marshall, D., Lloyd, A., Prosser, L. A., Regier, D. A., Johnson, F. R., & Mauskopf, J. (2011). Conjoint analysis applications in health - A checklist: A report of the ISPOR Good Research Practices for Conjoint Analysis Task Force. *Value in Health*, *14*(4), 403–413. https://doi.org/10.1016/j.jval.2010.11.013

Canelo-Aybar, C., Posso, M., Montero, N., Solà, I., Saz-Parkinson, Z., Duffy, S. W., Follmann, M., Gräwingholt, A., Giorgi Rossi, P., & Alonso-Coello, P. (2022). Benefits and harms of annual, biennial, or triennial breast cancer mammography screening for women at average risk of breast cancer: a systematic review for the European Commission Initiative on Breast Cancer (ECIBC). *British Journal of Cancer*, *126*(4), 673–688. https://doi.org/10.1038/s41416-021-01521-8

Davidson, A. S., Liao, X., Magee, D., Magee, B. D., & Magee, D. (2011). Attitudes of women in their forties toward the 2009 USPSTF mammogram guidelines: a randomized trial on the effects of media exposure. *AMERICAN JOURNAL OF OBSTETRICS AND GYNECOLOGY*, *205*(1), 30.e1-30.e7. https://doi.org/10.1016/j.ajog.2011.04.005

Evans, G. D., Warwick, J., Astley, S. M., Stavrinos, P., Sahin, S., Ingham, S., McBurney, H., Eckersley, B., Harvie, M., Wilson, M., Beetles, U., Warren, R., Hufton, A., Sergeant, J. C., Newman, W. G., Buchan, I., Cuzick, J., & Howell, A. (2012). Assessing individual breast cancer risk within the U.K. National Health Service Breast Screening Program: A new paradigm for cancer prevention. *Cancer Prevention Research*, *5*(7), 943–951. https://doi.org/10.1158/1940-6207.CAPR-11-0458

French, D., Astley, S., Brentnall, A., Cuzick, J., Dobrashian, R., Duffy, S., Gorman, L., Harrison, F., Harvie, M., Howell, A., Jerrison, A., Machin, M., Maxwell, A. J., Maxwell, A. J., Maxwell, A. J., McWilliams, L., Payne, K., Qureshi, N., Ruane, H., … Evans, D. G. (2020). What are the benefits and harms of risk stratified screening as part of the NHS breast screening Programme? Study protocol for a multi-site non-randomised comparison of BC-predict versus usual screening (NCT04359420). *BMC Cancer*, *20*(1). https://doi.org/10.1186/s12885-020-07054-2

Ghanouni, A., Sanderson, S. C., Pashayan, N., Renzi, C., von Wagner, C., & Waller, J. (2019). Attitudes towards risk-stratified breast cancer screening among women in England: A cross-sectional survey. *Journal of Medical Screening*. https://doi.org/10.1177/0969141319883662

Gigerenzer, G., Mata, J., & Frank, R. (2009). Public knowledge of benefits of breast and prostate cancer screening in Europe. *J Natl Cancer Inst*, *101*(17), 1216–1220. https://doi.org/10.1093/jnci/djp237

Jansen, J., & Houssami, N. (2018). Perceptions and misperceptions of overdetection of breast cancer. In *Translational Cancer Research* (Vol. 7, Issue 6, pp. 1347–1353). AME Publishing Company. https://doi.org/10.21037/tcr.2018.09.08

Kelley‐Jones, C., Scott, S., & Waller, J. (2021). Uk women’s views of the concepts of personalised breast cancer risk assessment and risk‐stratified breast screening: A qualitative interview study. *Cancers*, *13*(22). https://doi.org/10.3390/cancers13225813

Klarenbach, S., Sims-Jones, N., Lewin, G., Singh, H., Thériault, G., Tonelli, M., Doull, M., Courage, S., Garcia, A. J., Thombs, B. D., Klarenbach, S., Sims-Jones, N., Lewin, G., Singh, H., Thériault, G., Tonelli, M., Doull, M., Courage, S., & Jaramillo, A. (2018). Recommendations on screening for breast cancer in women aged 40-74 years who are not at increased risk for breast cancer. In *CMAJ* (Vol. 190, Issue 49, pp. E1441–E1451). Canadian Medical Association. https://doi.org/10.1503/cmaj.180463

Marmot, M. G., Altman, D. G., Cameron, D. A., Dewar, J. A., Thompson, S. G., & Wilcox, M. (2013). The benefits and harms of breast cancer screening: An independent review. *British Journal of Cancer*, *108*(11), 2205–2240. https://doi.org/10.1038/bjc.2013.177

MASCara, M., & Constantinou, C. (2021). *Global Perceptions of Women on Breast Cancer and Barriers to Screening*. https://doi.org/10.1007/s11912-021-01069-z/Published

Mathioudakis, A. G., Salakari, M., Pylkkanen, L., Saz‐Parkinson, Z., Bramesfeld, A., Deandrea, S., Lerda, D., Neamtiu, L., Pardo‐Hernandez, H., Solà, I., & Alonso‐Coello, P. (2019). Systematic review on women’s values and preferences concerning breast cancer screening and diagnostic services. *Psycho-Oncology*, *28*(5), pon.5041. https://doi.org/10.1002/pon.5041

McMenamin, M., Barry, H., Lennon, A. M., Purcell, H., Baum, M., Keegan, D., McDermott, E., O’Donoghue, D., Daly, L., & Mulcahy, H. (2005). A survey of breast cancer awareness and knowledge in a Western population: Lots of light but little illumination. *European Journal of Cancer*, *41*(3), 393–397. https://doi.org/10.1016/j.ejca.2004.11.015

McWilliams, L., Woof, V. G., Donnelly, L. S., Howell, A., Evans, D. G., & French, D. P. (2021). Extending screening intervals for women at low risk of breast cancer: do they find it acceptable? *BMC Cancer*, *21*(1). https://doi.org/10.1186/S12885-021-08347-W

Noguchi, N., Marinovich, M. L., Wylie, E. J., Lund, H. G., & Houssami, N. (2022). Evidence from a BreastScreen cohort does not support a longer inter-screen interval in women who have no conventional risk factors for breast cancer. *Breast*, *62*, 16–21. https://doi.org/10.1016/j.breast.2022.01.015

Pashayan, N., Antoniou, A. C., Lee, A., Wolfson, M., Chiquette, J., Eloy, L., Eisen, A., Stockley, T. L., Nabi, H., Brooks, J. D., Dorval, M., Easton, D. F., Knoppers, B. M., Chiarelli, A. M., & Simard, J. (2021a). Personalized Medicine Should Age-Dependent Absolute Risk Thresholds Be Used for Risk Stratification in Risk-Stratified Breast Cancer Screening? *J. Pers. Med*, *11*(9), 916. https://doi.org/10.3390/jpm11090916

Pashayan, N., Antoniou, A. C., Lee, A., Wolfson, M., Chiquette, J., Eloy, L., Eisen, A., Stockley, T. L., Nabi, H., Brooks, J. D., Dorval, M., Easton, D. F., Knoppers, B. M., Chiarelli, A. M., & Simard, J. (2021b). Personalized Medicine Should Age-Dependent Absolute Risk Thresholds Be Used for Risk Stratification in Risk-Stratified Breast Cancer Screening? *J. Pers. Med*, *11*(9), 916. https://doi.org/10.3390/jpm11090916

Pashayan, N., Morris, S., Gilbert, F. J., & Pharoah, P. D. P. (2018). Cost-effectiveness and Benefit-to-Harm Ratio of Risk-Stratified Screening for Breast Cancer A Life-Table Model. *JAMA Oncology*, *4*(11), 1504–1510. https://doi.org/10.1001/jamaoncol.2018.1901

Rainey, L., Van Der Waal, D., & Broeders, M. (2020). Dutch women’s intended participation in a risk-based breast cancer screening and prevention programme: A survey study identifying preferences, facilitators and barriers. In *BMC Cancer* (Vol. 20, Issue 1). BioMed Central Ltd. https://doi.org/10.1186/s12885-020-07464-2

Rouge-Bugat, M.-E., Balleyguier, C., Laurent, N., Dautreppe, A., Maillet, L., Simon, P., Fournet, P., Scellier, C., Menini, T., Darmon, E., Baron, C., Vissac-Sabatier, C., & Delaloge, S. (2022). MyPeBS International randomized study comparing personalised, risk-stratified to standard breast cancer screening in women aged 40–70: Focus on recruitment strategy in France. *La Presse Médicale Open*, *3*, 100022. https://doi.org/10.1016/j.lpmope.2022.100022

Rozbroj, T., Haas, R., O’Connor, D. A., Thomas, R., McCaffery, K., Carter, S., & Buchbinder, R. (2020). How do patients and the public understand overtesting and overdiagnosis? A protocol for a thematic meta-synthesis of qualitative research. *BMJ Open*, *10*(7). https://doi.org/10.1136/BMJOPEN-2020-037283

Rozbroj, T., Haas, R., O’Connor, D., Carter, S. M., McCaffery, K., Thomas, R., Donovan, J., & Buchbinder, R. (2021). How do people understand overtesting and overdiagnosis? Systematic review and meta-synthesis of qualitative research. In *Social Science and Medicine* (Vol. 285, p. 114255). Pergamon. https://doi.org/10.1016/j.socscimed.2021.114255

Schünemann, H. J., Lerda, D., Quinn, C., Follmann, M., Alonso-Coello, P., Rossi, P. G., Lebeau, A., Nyström, L., Broeders, M., Ioannidou-Mouzaka, L., Duffy, S. W., Borisch, B., Fitzpatrick, P., Hofvind, S., Castells, X., Giordano, L., Canelo-Aybar, C., Warman, S., Mansel, R., … Saz-Parkinson, Z. (2020). Breast cancer screening and diagnosis: A synopsis of the european breast guidelines. *Annals of Internal Medicine*, *172*(1), 46–56. https://doi.org/10.7326/M19-2125

Seaman, K., Dzidic, P. L., Castell, E., Saunders, C., & Breen, L. J. (2018). A Systematic Review of Women’s Knowledge of Screening Mammography. In *Breast* (Vol. 42, pp. 81–93). Churchill Livingstone. https://doi.org/10.1016/j.breast.2018.08.102

Silverman, E., Woloshin, S., Schwartz, L. M., Byram, S. J., Welch, H. G., & Fischhoff, B. (2001). Women’s views on breast cancer risk and screening mammography: A qualitative interview study. *Medical Decision Making*, *21*(3), 231–240. https://doi.org/10.1177/0272989X0102100308

Trevena, L. J., Bonner, C., Okan, Y., Peters, E., Gaissmaier, W., Han, P. K. J., Ozanne, E., Timmermans, D., & Zikmund-Fisher, B. J. (2021). Current Challenges When Using Numbers in Patient Decision Aids: Advanced Concepts. *Medical Decision Making*, *41*(7), 834–847. https://doi.org/10.1177/0272989X21996342

UNICANCER. (2017). *20170409_MyPeBS_clinical_trial_master_clean*.

Van Den Broek, J. J., Schechter, C. B., Van Ravesteyn, N. T., Janssens, A. C. J. W., Wolfson, M. C., Trentham-Dietz, A., Simard, J., Easton, D. F., Mandelblatt, J. S., Kraft, P., & De Koning, H. J. (2021). Personalizing Breast Cancer Screening Based on Polygenic Risk and Family History. *JNCI: Journal of the National Cancer Institute*, *113*(4), 434–442. https://doi.org/10.1093/JNCI/DJAA127

Wegwarth, O., Widschwendter, M., Cibula, D., Sundström, K., Portuesi, R., Lein, I., & Rebitschek, F. G. (2018). What do European women know about their female cancer risks and cancer screening? A cross-sectional online intervention survey in five European countries. *BMJ Open*, *8*, 23789. https://doi.org/10.1136/bmjopen-2018-023789

Witteman, H. O., Ndjaboue, R., Vaisson, G., Dansokho, S. C., Arnold, B., Bridges, J. F. P., Comeau, S., Fagerlin, A., Gavaruzzi, T., Marcoux, M., Pieterse, A., Pignone, M., Provencher, T., Racine, C., Regier, D., Rochefort-Brihay, C., Thokala, P., Weernink, M., White, D. B., … Jansen, J. (2021). Clarifying Values: An Updated and Expanded Systematic Review and Meta-Analysis. In *Medical Decision Making* (Vol. 41, Issue 7, pp. 801–820). https://doi.org/10.1177/0272989X211037946

Woof, V. G., McWilliams, L., Donnelly, L. S., Howell, A., Evans, D. G., Maxwell, A. J., & French, D. P. (2021). Introducing a low-risk breast screening pathway into the NHS Breast Screening Programme: Views from healthcare professionals who are delivering risk-stratified screening. *Women’s Health*, *17*. https://doi.org/10.1177/17455065211009746

Zielonke, N., Kregting, L. M., Heijnsdijk, E. A. M., Veerus, P., Heinävaara, S., McKee, M., de Kok, I. M. C. M., de Koning, H. J., van Ravesteyn, N. T., Gredinger, G., De Brabander, I., Arbyn, M., Simoens, C., Martens, P., Candeur, M., Arbyn, M., Simoens, C., Burrion, J. B., Dimitrov, P., … Latinovic, R. (2021). The potential of breast cancer screening in Europe. *International Journal of Cancer*, *148*(2), 406–418. https://doi.org/10.1002/ijc.33204

## **S2: *Experimental design***

| Choice set | start | end | int | riskdie | odx | alt | block |
| --- | --- | --- | --- | --- | --- | --- | --- |
| 1a | 55 | 70 | 7 | 24 | 10 | 1 | 2 |
| 1b | 60 | 65 | 3 | 26 | 2 | 2 | 2 |
| 2a | 50 | 65 | 5 | 26 | 6 | 1 | 1 |
| 2b | 55 | 70 | 3 | 25 | 2 | 2 | 1 |
| 3a | 60 | 65 | 3 | 24 | 6 | 1 | 1 |
| 3b | 50 | 70 | 7 | 26 | 10 | 2 | 1 |
| 4a | 50 | 65 | 7 | 25 | 2 | 1 | 2 |
| 4b | 55 | 70 | 5 | 26 | 6 | 2 | 2 |
| 5a | 60 | 70 | 3 | 26 | 10 | 1 | 2 |
| 5b | 50 | 65 | 5 | 24 | 2 | 2 | 2 |
| 6a | 60 | 65 | 5 | 26 | 2 | 1 | 2 |
| 6b | 50 | 70 | 3 | 24 | 6 | 2 | 2 |
| 7a | 60 | 70 | 5 | 24 | 2 | 1 | 2 |
| 7b | 55 | 65 | 3 | 27 | 10 | 2 | 2 |
| 8a | 60 | 65 | 3 | 27 | 6 | 1 | 1 |
| 8b | 50 | 70 | 5 | 26 | 2 | 2 | 1 |
| 9a | 55 | 65 | 5 | 27 | 2 | 1 | 1 |
| 9b | 50 | 70 | 7 | 25 | 10 | 2 | 1 |
| 10a | 60 | 65 | 5 | 24 | 10 | 1 | 1 |
| 10b | 50 | 70 | 3 | 27 | 2 | 2 | 1 |
| 11a | 55 | 70 | 7 | 24 | 2 | 1 | 2 |
| 11b | 50 | 65 | 5 | 25 | 6 | 2 | 2 |
| 12a | 55 | 65 | 5 | 27 | 10 | 1 | 2 |
| 12b | 60 | 70 | 3 | 26 | 6 | 2 | 2 |
| 13a | 55 | 70 | 5 | 25 | 6 | 1 | 1 |
| 13b | 50 | 65 | 3 | 24 | 10 | 2 | 1 |
| 14a | 55 | 65 | 7 | 26 | 6 | 1 | 1 |
| 14b | 60 | 70 | 5 | 25 | 10 | 2 | 1 |
| 15a | 60 | 70 | 5 | 27 | 10 | 1 | 1 |
| 15b | 55 | 65 | 7 | 25 | 6 | 2 | 1 |
| 16a | 55 | 65 | 3 | 25 | 10 | 1 | 2 |
| 16b | 60 | 70 | 7 | 27 | 6 | 2 | 2 |

## ***S3*. DCE survey template**

**Women’s views on NHS breast screening if assessed to be at low breast cancer risk**

[Section 1: Participant information & consent]

We would like to invite you to participate in this study which aims to understand women’s views on breast cancer screening. Before you decide whether you want to take part in the main survey, it is important for you to understand why the study is being done and what your participation will involve. Please take the time to read the following information carefully and discuss it with others if you wish. Please do not hesitate to contact us if there is anything that is not clear or you would like more information.

**What is the purpose of the study?**

Today, scientists know how to identify a woman’s individual level of risk for developing breast cancer. This additional information may allow us to improve NHS breast screening by tailoring screening to breast cancer risk. The purpose of this study is to gather your responses to different screening options that could benefit women identified as having low risk of developing breast cancer.

**Why have I been invited to take part?**

You have been invited to take part because, as a woman aged between 40 and 70, you may have, or will soon have some experience of being offered breast screening.

At the start of the survey, we will ask you some questions to make sure that it is appropriate for you to take part in the study. We are looking for individuals who are:

- Aged 40-70

You are **not** eligible to take part if you:

- Have been diagnosed with breast cancer
- Have been identified as having a high risk of breast cancer and are invited to have annual NHS breast screening.

**What will happen if I take part?**

If you agree to take part, you will complete the survey anonymously which means that nobody, including the researchers, will be aware of your identity. No one will be able to connect you to the answers you provide, even indirectly. The survey will ask a series of questions to explore your thoughts on breast screening options for women identified as having low risk of developing breast cancer. There are no right or wrong answers, we just want to see what you think. The online survey will take about 20-25 minutes to complete and will involve reading some information as well as answering questions.

**Do I have to take part?**

Participation is completely voluntary. You should only take part if you want to and choosing not to take part will not disadvantage you in any way. If you choose to take part, we will ask you to provide your consent. To do this, you will be asked to indicate that you have read and understood the information provided and that you consent to your anonymous data being used for the purposes explained. You can withdraw from the study at any time without giving a reason by closing the browser. However, once you start the survey, your responses will automatically be stored even if you close your browser and stop the survey at any point. This means that if you start the survey, you can drop out, but we will keep your responses to any questions you have already answered.

**Data handling and confidentiality**

The research is anonymous. This means that nobody, including the researchers, will be aware of your identity, and that nobody will be able to connect you to the answers you provide, even indirectly. The online panel company, Savanta, use a unique ID number to see which of their panel members take part in which studies.

When you complete the survey, we will send your unique ID number to Savanta so they can match this with your contact details to process a financial reward for your time and effort. Once we have sent your ID number to Savanta, we will securely delete it from our data set. At no point will your contact details be shared with us. Savanta will not have access to any of your questionnaire responses.

All of your answers will be treated as confidential, and you will not be identified in any research outputs or publications. The data controller for this study will be King’s College London (KCL). Research is a task that the University carries out in the public interest. Anonymous data will be kept for a minimum period of six years after the completion of the study and may be shared with other researchers. The data will be stored on secure internal servers within KCL.

**Data Protection Statement**

Your data will be processed in accordance with the General Data Protection Regulation 2016 (GDPR). If you would like more information about how your data will be processed in accordance with GDPR please visit the link below: [https://www.kcl.ac.uk/research/support/research-ethics/kings-college-london-statement-on-use-of-personal-data-in-research](about:blank).

**How is the study being funded?**

This study is being funded by Breast Cancer Now (https://breastcancernow.org/).

**What will happen to the results of the study?**

The results of the study will be analysed and written up for publication in academic journals, presented at conferences and will form part of a PhD thesis. Findings may also be published in the King’s College London Cancer Prevention Group’s blog ([https://blogs.kcl.ac.uk/cancerprevention](about:blank)) and on social media pages (e.g., Twitter).

**Potential benefits and risks**

If you complete the survey, you will receive the usual incentive from Savanta, as described in the email that they have sent you. There are no other direct benefits of taking part, but the information that you provide may help shape the future direction of the NHS breast cancer screening programme.

We do not anticipate any risks to taking part. However, we understand that some people may find it upsetting to answer questions about breast cancer or previous breast screening experiences. You will be given the option of choosing “prefer not to say” for several questions. In addition, we will provide you with contact details for further sources of information and support, should you need it.

**Who should I contact for further information?**

If you have any questions or require more information about this study, please contact Charlotte Jones ([charlotte.kelley_jones@kcl.ac.uk](about:blank)).

**What if I have further questions, or if something goes wrong?**

If this study has harmed you in any way or if you wish to make a complaint about the conduct of the study you can contact the Ethics Subcommittee of Biomedical & Health Science, Dentistry, Medicine and Natural & Mathematical Science at King's College London (bdm@kcl.ac.uk" bdm@kcl.ac.uk).

**Thank you for reading this information page and for considering taking part in this research.**

I have read and understood the information provided and consent to my anonymous data being used for the purposes explained.

**(1.1)**

**Yes**

**No**  **[route out to: Thank you for your interest in this study.]**

**[Section 2: Some background about you**

We’d like to start by asking you a few questions to make sure you are eligible for the study

**(2.1.) How old are you?**

39 years or younger **[route out to: Thank you for taking the time to look at this study. Unfortunately, you are not eligible to participate as we are only inviting women aged between 40 and 70 years.**

40-45 years

46-50 years

51-55 years

56-60 years

61-65 years

66-70 years

71 years or older **[route out to: Many thanks for taking the time to look at this study. Unfortunately, you are not eligible to participate as we are only inviting women aged between 40 and 70 years.]**

**(2.2) What is your exact age?**

o Prefer not to say

| \| **Which ONE of the following best describes the occupation of the main income earner in your household? If you or the main income earner are self-employed, please tick the option that most relates to the type of work you/they do for the company(s) you/they work for.** \| \| --- \|  \| Higher managerial/ professional/ administrative (e.g., Doctor, Solicitor, Board Director in a large organisation 200+ employees, top level civil servant/public service employee etc.) \| \| --- \| \| Intermediate managerial/ professional/ administrative (e.g., Newly qualified (under 3 years) doctor, Solicitor, Board director of small organisation, middle manager in large organisation, principle officer in civil service/local government etc.) \| \| Supervisor; clerical; junior managerial administrative or professional (e.g., Office worker, Student Doctor, Foreman with 25+ employees, salesperson, etc.) \| \| Skilled manual worker (e.g., Bricklayer, Carpenter, Plumber, Painter, Bus/ Ambulance Driver, HGV driver, pub/bar worker etc.) \| \| Semi or unskilled manual worker (e.g., Caretaker, Park keeper, non-HGV driver, shop assistant etc.) \| \| Student \| \| Unemployed or not working due to long-term sickness \| \| Casual worker - not in permanent employment \| \| Full-time carer of other household member \| \| Retired \| \| Rather not say \| |
| --- | --- | --- | --- | --- | --- | --- | --- | --- | --- | --- | --- | --- |

[Inserted by Accent Marketing & Research Ltd., to determine SES]

**(2.3) Have you ever been diagnosed with breast cancer?**

Yes **[route out to: Many thanks for taking the time to look at this study. Unfortunately, you are not eligible to participate as we are only inviting women with no previous diagnosis of breast cancer.]**

No

**(2.4) Have you been identified as being at high risk of breast cancer? (e.g., have genetic variants BRCA1/2 and/or have been recommended to have annual NHS breast screening).**

Yes **[route out to: Many thanks for taking the time to look at this study. Unfortunately, you are not eligible to participate as this survey focuses on low-risk breast screening options.]**

No

Not sure **[route out to: Many thanks for taking the time to look at this study. Unfortunately, you are not eligible to participate as this study focuses on low-risk breast screening options.**

**(2.5) What is the highest qualification you have achieved?**

No formal qualifications

O’ Level or GCSE equivalent

A’ Levels or Higher’s

Higher education below degree

Bachelor’s degree (BA, BSc)

Further degree or higher, e.g*.*, Masters, PhD etc.

Still studying

Other

**(2.6) What is your relationship status?**

Single

Married, civil partnership or living with a partner

Divorced or separated

Widowed

Other

Prefer not to say

**(2.7) What is your ethnic group?**

Please choose one of the following options that best describes your ethnic group or background.

White

Includes English, Welsh, Scottish, Northern Irish, British, Irish Gypsy or Irish traveller and any other White background, e.g., White European

Mixed / multiple ethnic groups

Includes White and and Black Caribbean, White and Black African, White and Asian and any other mixed/multiple ethnic background

- **Asian /Asian British**

Includes Indian**,** Pakistani, Bangladeshi, Chinese and any other Asian background

- **Black / Black British**

Includes Black African, Black Caribbean, Black British and any other Black/African/Caribbean background

- **Other ethnic group**

Includes Arab and any other ethnic group, please describe

- Prefer not to say

**[Section 3: Your experience of breast screening**

We would now like to ask you a little bit about your experience of breast screening. If you are under 50 years old and not yet eligible for breast screening please just tick the box stating that you have never been invited for breast screening.

**(3.1) Which of the following best describes your experience of NHS breast screening?**

I have never been invited for breast screening (e.g., because you are under 50 and not yet eligible for breast screening)

I have been invited but have never attended

I have attended before but have sometimes delayed or missed my screening appointment

I have always attended when invited

Prefer not to say

**(3.2) How would you rate your chances of developing breast cancer, compared with other women your age?**

Much lower

A little lower

About the same

A little higher

Much higher

Don’t know

**(3.3) How anxious do you feel when you think about breast cancer?**

Not at all

Slightly

Quite a bit

Extremely

**(3.4) How often do you worry about your chance of getting breast cancer?**

Never

Occasionally

Sometimes

Often

Very often

**(3.5)** **Has anyone in your family or any of your friends had breast cancer?**

Please tick any of the boxes that apply

Close family member

Other family member

Partner

Close friend

Other friend/colleague

No-one I know has had breast cancer

**[Section 4: Background information]**

Before moving on to the main part of the survey, it is important that you take your time to read the following background information.

**What is the NHS breast screening programme and how does it work?**

**Breast screening** uses an X-ray test called a mammogram to check the breast for signs of cancer. It can spot cancers that are too small to see or feel.

The **UK NHS breast screening programme** offers breast screening every three years to **all** women **aged** 50 to 70 years. All health screening programmes (including breast screening) have **benefits** **and** **harms.**

**What is the main benefit of breast screening?**

- Breast screening can **save lives** from **breast cancer** by **diagnosing it earlier** when it is **easier** to treat.
- Breast screening is thought to **save about 1 life from breast cancer for every 200 women** who are screened every 3 years from the age of 50-70.

**What is the main harm of breast screening?**

- Some women will be diagnosed and treated for breast cancers that would never have been found without screening and would **never have become life-threatening**.
- Doctors cannot always tell whether a breast cancer that is diagnosed will go on to be life-threatening or not, so they offer treatment to **all** women with a breast cancer.
- This means that some women will be **offered treatment that they do not need**. This is known as **over-detection** or overdiagnosis.
- About **3 in** **every 200 women screened every 3 years from the age of 50-70 are diagnosed with a breast cancer that would never have become life-threatening**.

**How can the NHS breast screening programme be improved?**

At the moment, **all** women in the UK are invited for breast screening every three years on the basis of their age (50 to 70). There is growing interest in inviting women for screening based on both their age and additional risk information.

**What types of additional risk information can be measured?**

**
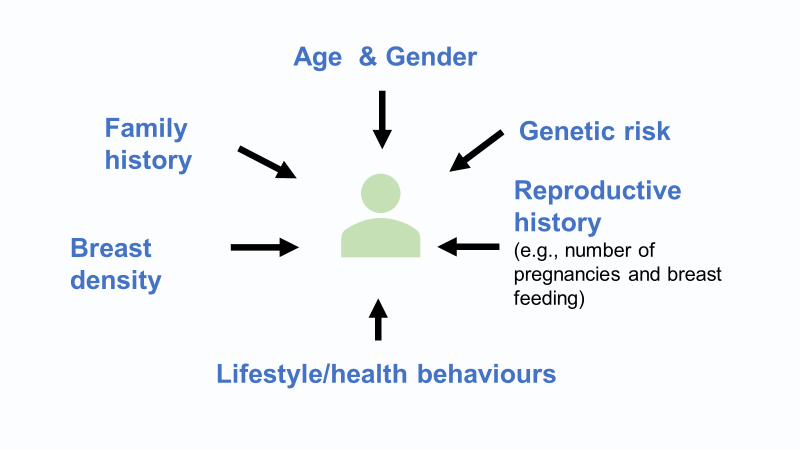
**

The figure above shows the types of information that can be used to assess a woman’s breast cancer risk.

**How would this personal risk information be used?**

Doctors will be able to use this information to calculate whether women have a higher or lower than average personal risk of developing breast cancer.

**How would including a woman’s personal risk of developing breast cancer change the breast screening programme?**

- Having breast screening every 3 years from age 50 to 70 may not be enough for women at **high risk** and may be too much for women at **low risk**.
- A woman’s risk information could be used to offer her the right amount of breast screening for her level of risk – more screening for women at high risk and less screening for women at low risk.
- This would be called **risk-based breast screening.**
- This approach could increase the **benefit** of screening for those at high risk and reduce the possible **harm** of screening for those at low risk.

**What would a risk-based breast screening programme look like?**

The diagram shows how women would be sorted into risk groups (low, average, moderate and high). Groups with higher or lower than average risk would be offered more or less screening, depending on their risk level. In this example, the risk of 20 women is assessed and 4 are classified as being at low risk, 10 at average risk, 4 at moderate risk and 2 at high risk.


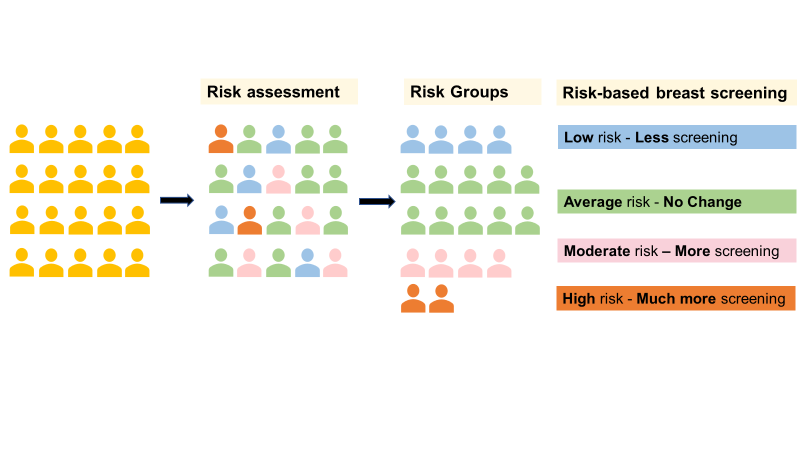


**[Section 5: How the next part of the survey works**

In this survey, we would like you to think about the group of women who are identified as being at **low risk** of breast cancer as part of a **risk-based breast screening** programme**.** We are interested in your views on how much less screening this group could be offered.

During this survey, we will ask you to make a series of choices between **two possible screening options** for women identified to be at low risk of developing breast cancer in their lifetime. Although a woman’s risk level might change over time, for the purpose of this task, we would like you to assume that these women will **remain at low risk throughout the period of breast screening eligibility**.

Each screening option will be described according to five features (examples in brackets):

- **Screening frequency** (e.g., screening every 3 years)
- **Age when screening will start** (e.g., screening starts at age 50 years)
- **Age when screening will stop** (e.g., screening stops at age 70 years)
- **Risk of over-detection** (picking up a breast cancer that would never have caused harm) (e.g., 5 for every 1000 women screened)
- **Risk of dying from breast cancer** (e.g., 2 for every 1000 women screened)

In each case, there will also be a **no screening** option.

To familiarise yourself with this idea, please try this practice question. As there are no right or wrong answers, we’d like you to use your intuition. (Feel free to follow the links to further information for any of the terms or features you are unsure about).


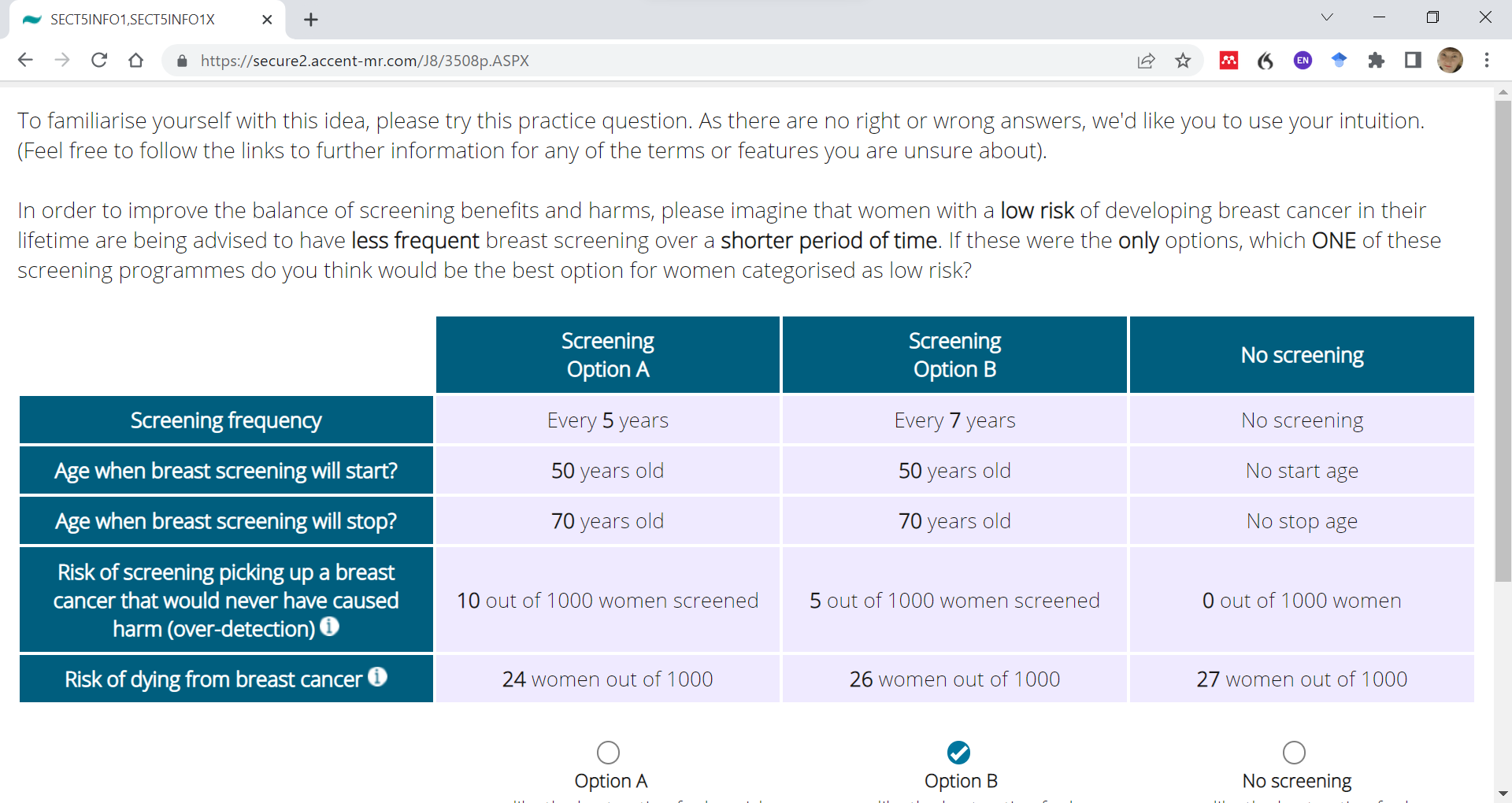


**Thank you for completing the practice question.**

**Section 6: Choice tasks: Randomised choice sets x 8 & survey experience**

**6.1- 6.8 :** Sample of how the 8 choice tasks were presented to a participant.


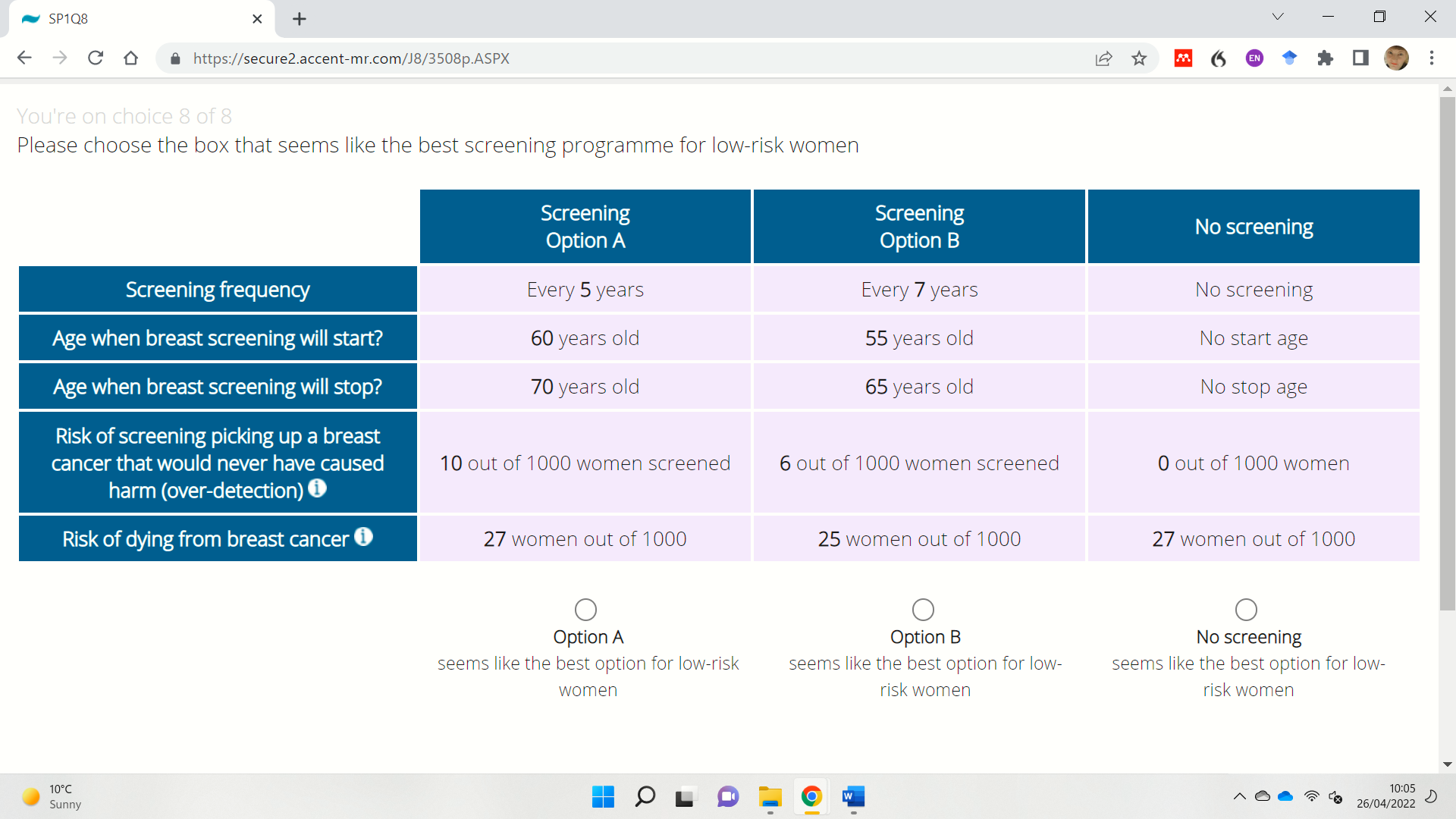


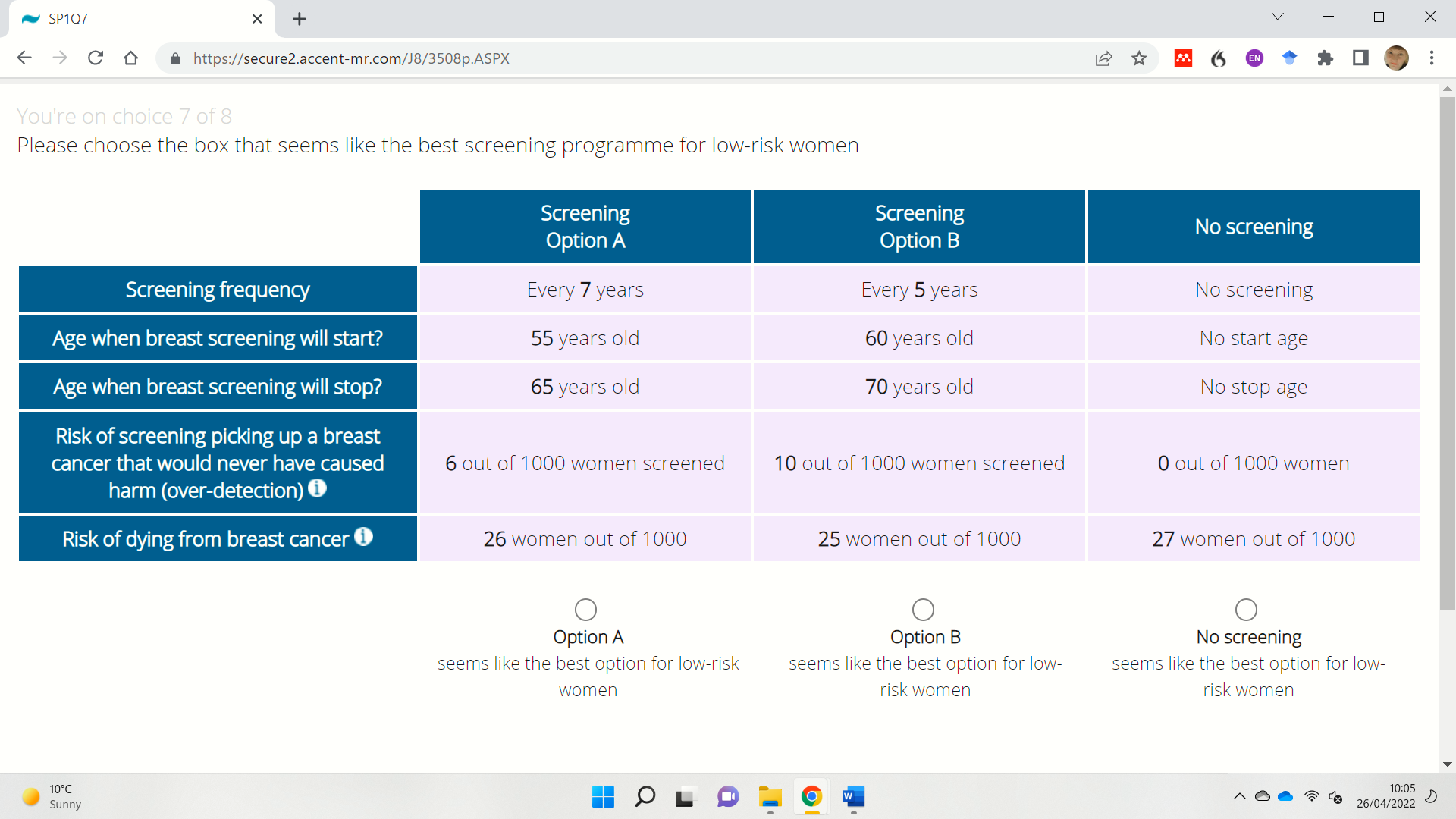


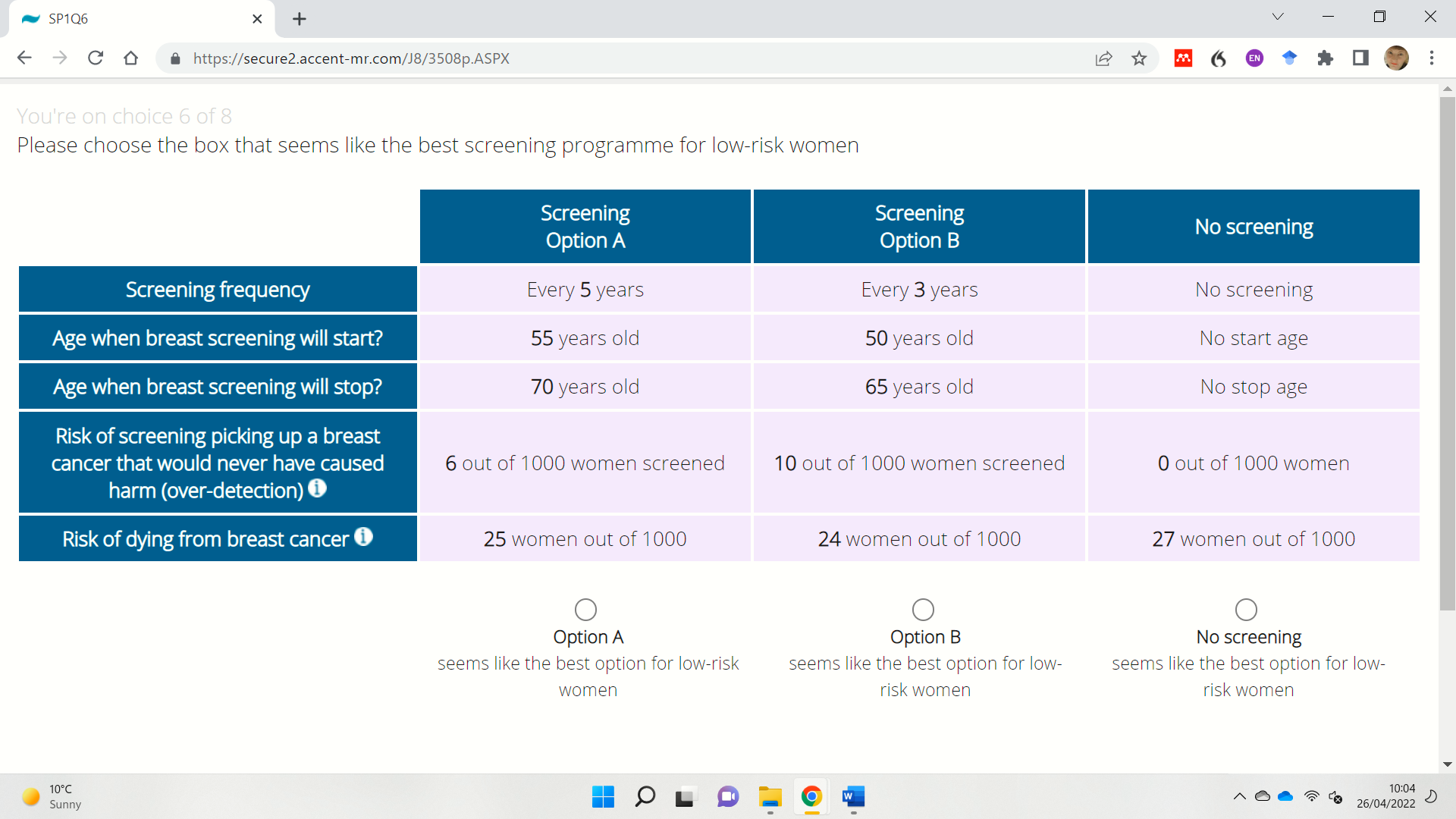


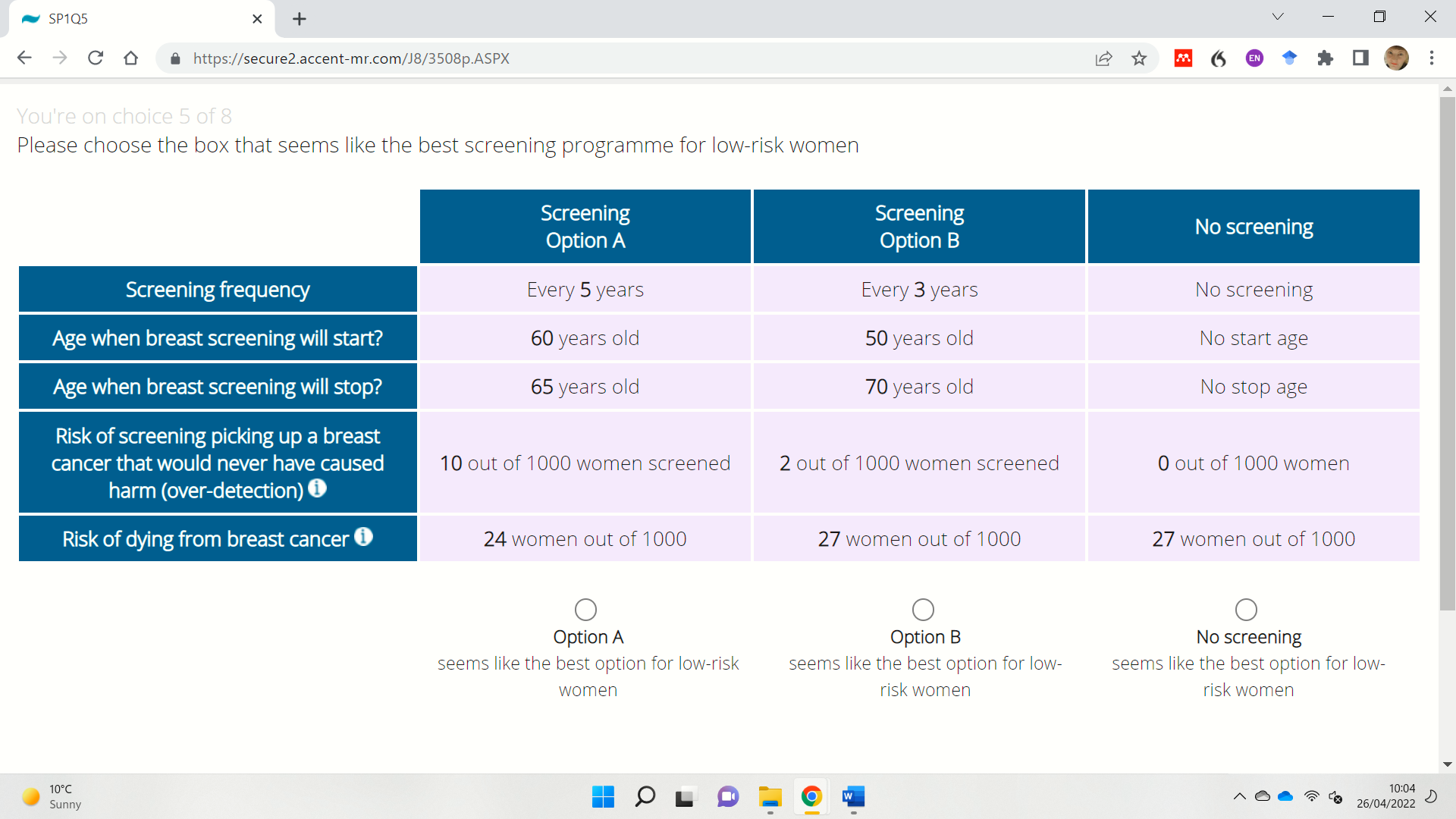


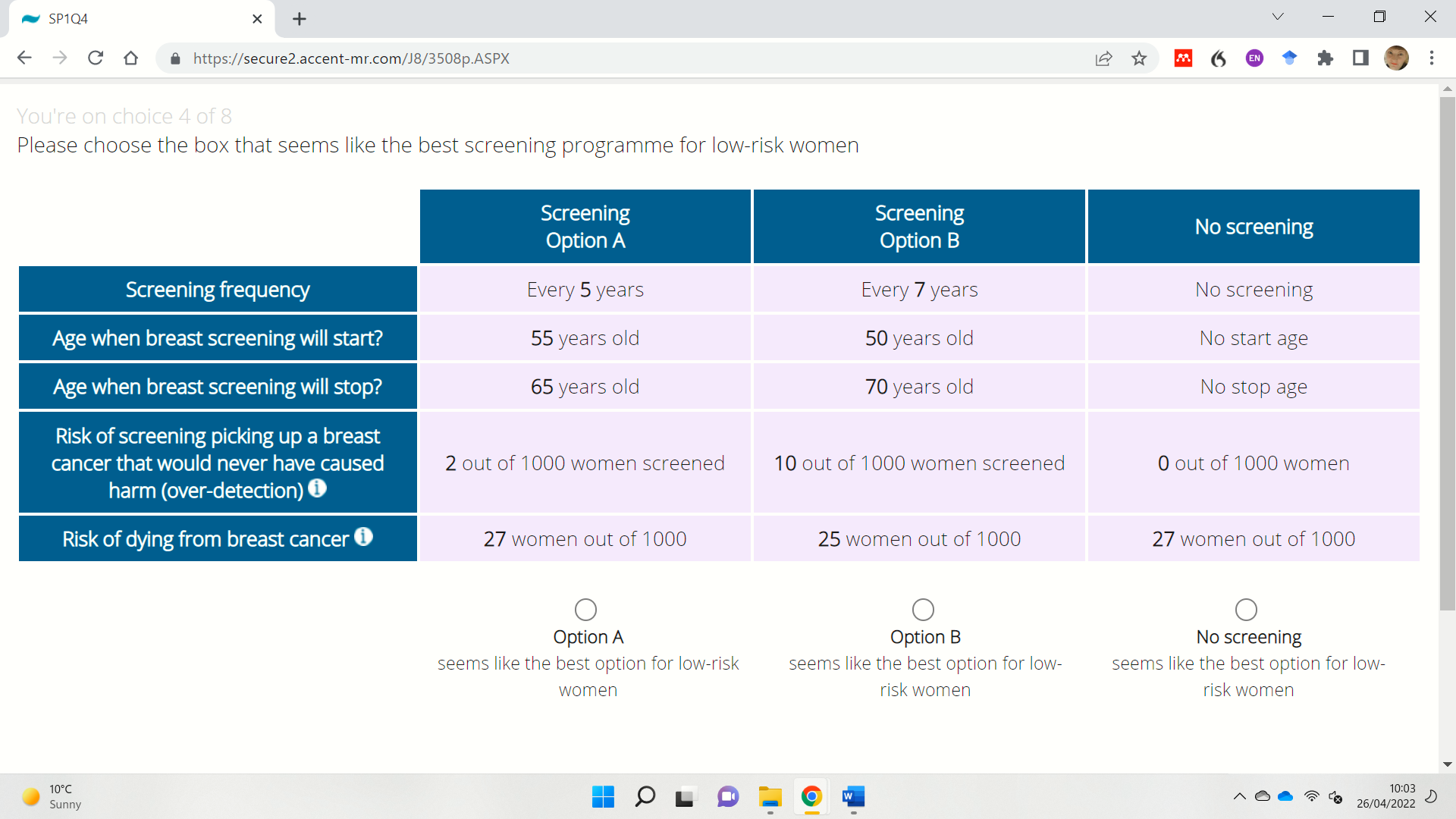


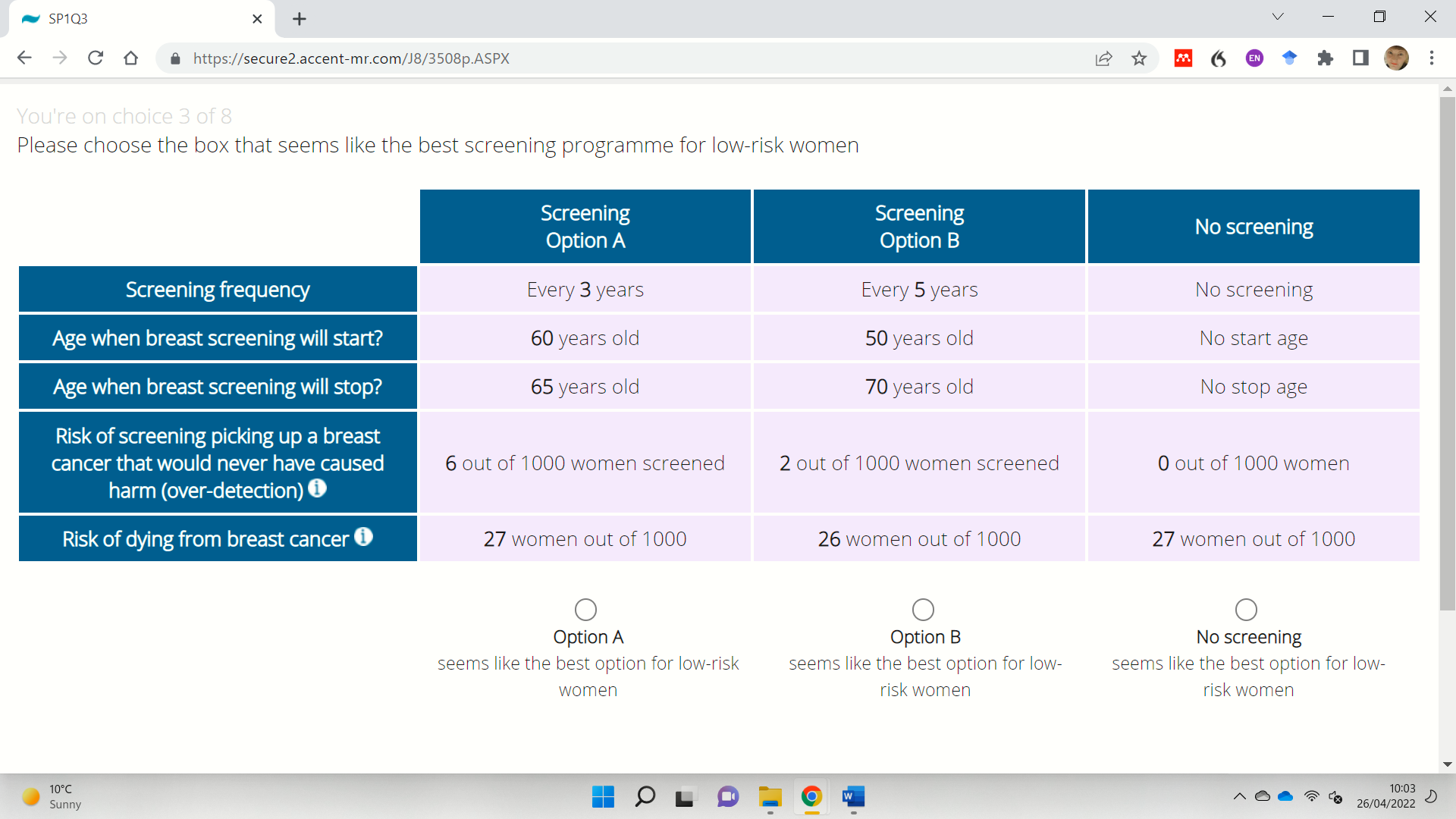


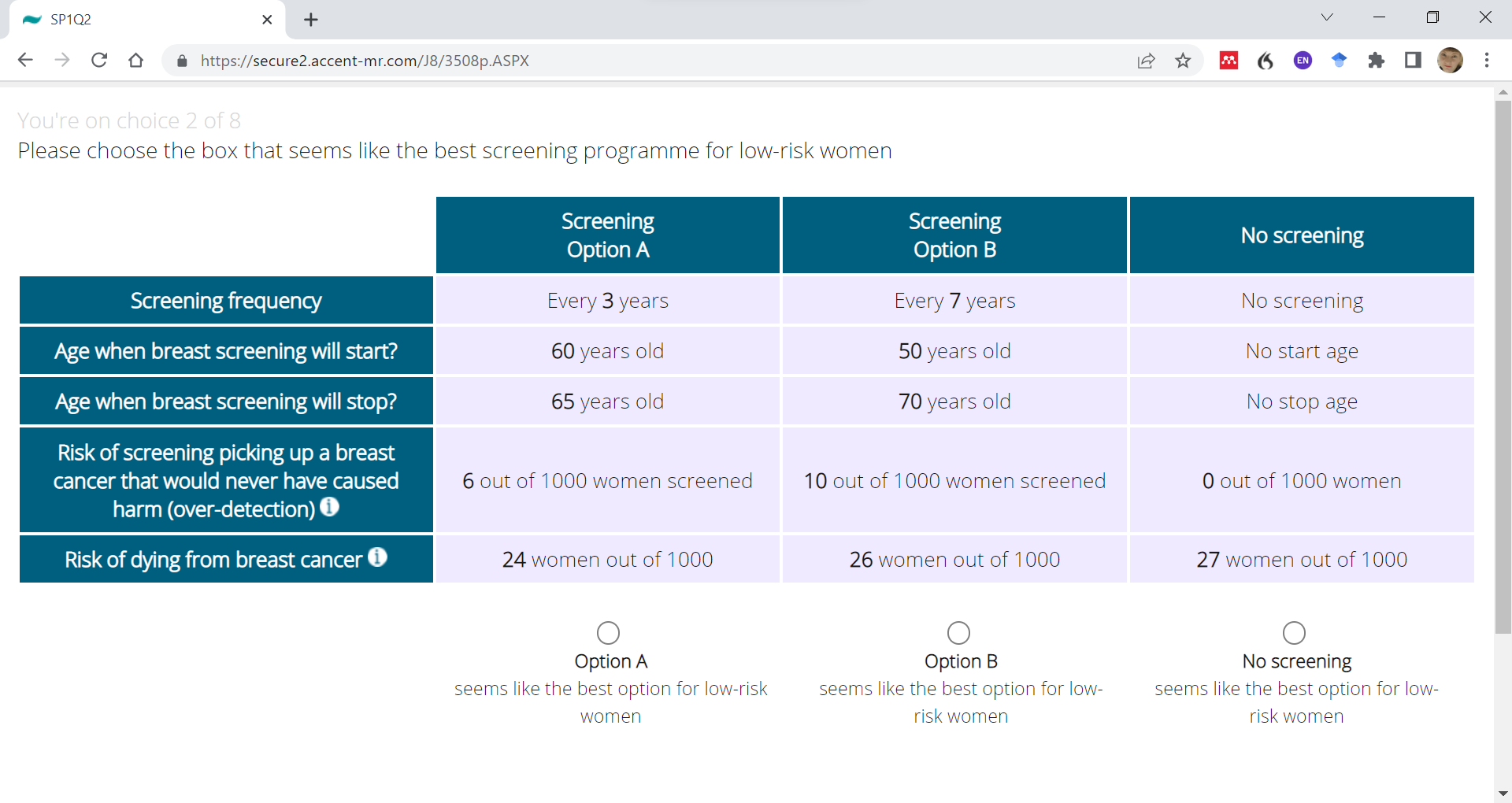


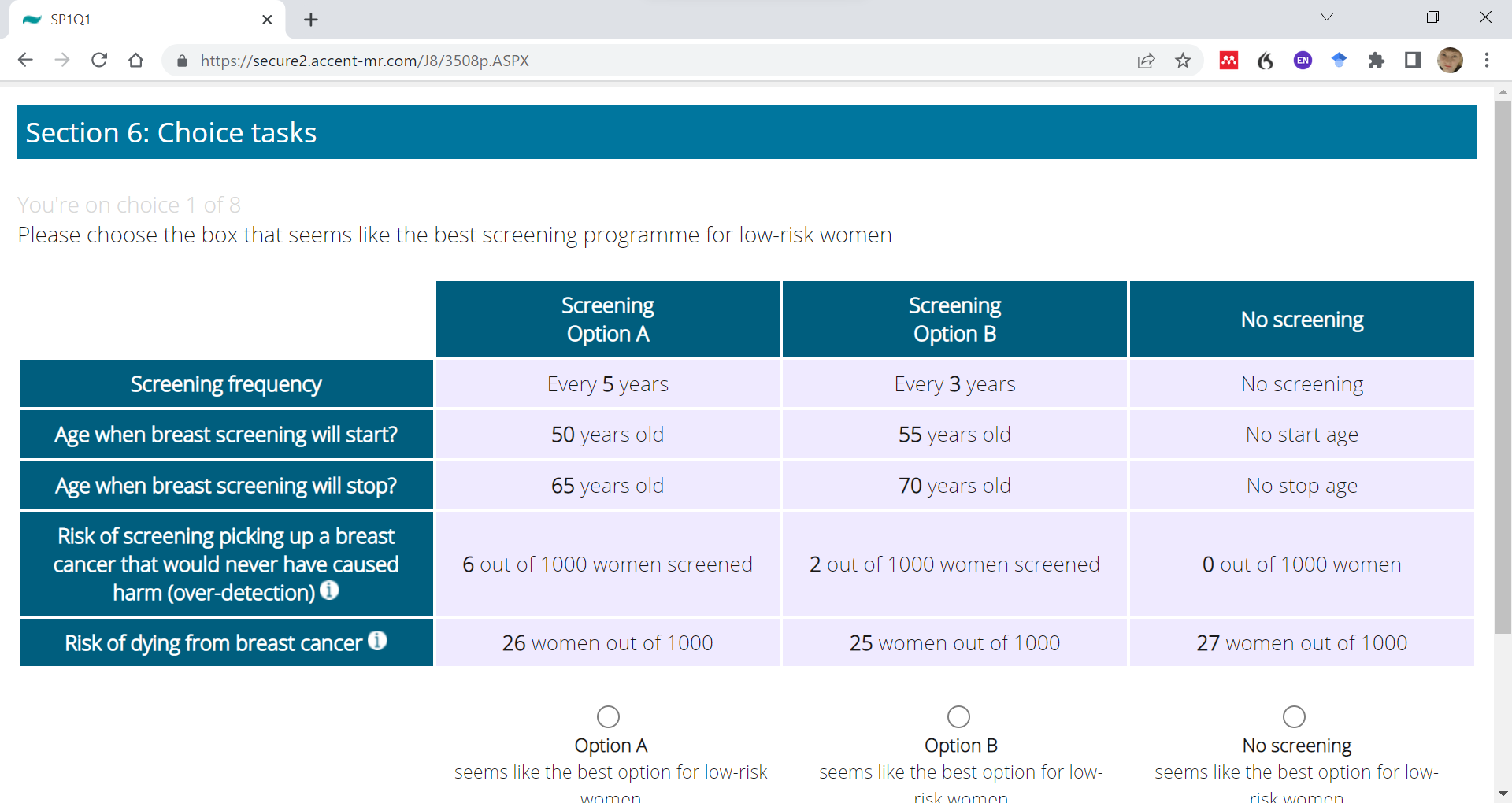


**Thank you for providing your choices. We would be interested to have some feedback about your experience of completing these choice tasks.**

**6.9** **How easy or difficult did you find it to understand what we were asking you to do in the choice tasks?**

Very easy

Quite easy

Neither easy nor difficult

Quite difficult

Very difficult

**6.10**. **How easy or difficult did you find making the choices between the three options?**

Very easy

Quite easy

Neither easy nor difficult

Quite difficult

Very difficult

**[Section 7: Final questions about you**

Thank you! You have reached the final set of questions. Again, there are no right or wrong answers here, we are just interested in exploring your thoughts and attitudes.

**7.1. Before taking part in this study, were you aware that breast screening could pick up a breast cancer that may never have caused harm (over-detection)?**

Yes (route to 7.2)

No (route to 7.3)

Not sure (route to 7.3)

**7.2. If YES, how did you learn about the risk of breast screening picking up a breast cancer that may never have caused harm?**

NHS Breast Screening leaflet

Health professional, e.g., your GP, practice nurse or breast screening technician

Word-of-mouth

Social media

Other media – e.g., newspaper, magazines, or TV

Other:

Not sure/can’t remember

7.3. In general, how easy or hard did you find it to understand the information on over-detection that was given at the start of the survey?

Very easy

Easy

Hard

Very hard

[Section 8 Further information/support]

**Thank you for completing this survey.**

Thank you for taking the time to complete the survey. Your answers are very helpful for our research.

The key aims of this study were:

1. To examine preferences for breast screening approaches for women identified as being at low-risk in a risk-based breast screening programme.

2. To understand how your preferences may vary with your general thoughts and feelings about breast cancer and the NHS breast screening programme.

Please note that the current evidence suggests that breast screening reduces the number of deaths from breast cancer by about 1,300 a year in the UK ([https://www.cancerresearchuk.org](about:blank)) and that the screening scenarios in this survey are **not current breast screening policy**. If you are worried about any of the things you have read about in this survey, please speak with your GP, or contact the research team.

Please find below some organisations which you can contact for further information or support:

**Breast Cancer Now:** [https://breastcancernow.org/](about:blank)**.** This research and care charity has a forum to share any experiences, concerns and will help you to access support ([https://forum.breastcancernow.org/](about:blank)). Alternatively, you can talk to one of their breast care nurses on **0808 800 6000**

For further information about the NHS breast screening programme, this link contains a video explaining the benefits and harms of breast screening [https://www.nhs.uk/conditions/breast-cancer-screening/](about:blank)**.**

You can also download the NHS breast screening leaflet here: [https://www.gov.uk/government/publications/breast-screening-helping-women-decide](about:blank).

If you have any questions about the study, you can contact the research team – Charlotte Jones ([charlotte.kelley_jones@kcl.ac.uk](about:blank)).

____________

**The following pop-ups will be embedded in the on-line survey as hover buttons:**

**Age:** A woman’s age is the most important measure of risk for breast cancer. The risk of getting breast cancer increases as you get older. About **4** out of **5** breast cancers are found in women over 50 years old.

**Balance of benefits to harms:** All health screening programmes have benefits and harms. The main benefit of breast screening is the early detection of breast cancer which may be more treatable as this has been diagnosed at an early stage. The harms of breast screening include over-detection which can happen when women are diagnosed and treated for a breast cancer that would never have harmed them. Current evidence suggests that the benefits of breast screening outweigh the harms. However, doctors are aware that this balance can be improved. Hence, the interest in the idea of using personal risk information to help improve the balance of benefit to harm for individual women.

**Benefit of breast screening:** The main benefit of breast screening is the early detection of breast cancer which may be more treatable as this has been diagnosed at an early stage.

**Harm of breast screening:** Over-detection is the main harm of breast screening which can happen when women are diagnosed and treated for a breast cancer that would never have harmed them.

**Breast cancer:** Breast cancer starts in the breast tissue, most commonly in the cells that line the milk ducts of the breast. There are many different types of breast cancers and all of these can behave in different ways with some faster growing and more aggressive than others. Although breast cancer is the most common type of cancer for women in the UK, survival from the disease is improving all the time due to advanced treatments. Today, about **3** out of **4** women diagnosed with breast cancer are alive 10 years later.

**Breast density:** Breast cancer risk is higher in women with the most dense breast tissue compared to less dense tissue. Women with dense breast tissue have less fat and more breast cells and connective tissue in their breasts.

**Breast screening:** Breast screening uses an X-ray test called a mammogram to check the breasts for early signs of breast cancer. It can spot cancers that are too small to see or feel.

**Family history:** It is thought that women with first degree relatives (e.g., a mother or a sister) who have been diagnosed with breast cancer may be at higher risk of developing the disease. You would be able to self-report any family history information by completing a short survey.

**Gender:** Just being a woman is the biggest risk factor for developing breast cancer. While men do develop breast cancer, they account for less than 1% of all new breast cancer diagnoses.

**Genetic risk:** Scientists have now identified many individual genetic variants that can increase the risk of breast cancer. Women will be able to have their genetic risk assessed by providing a blood or saliva sample.

**Lifestyle/health behaviours**: Lack of physical exercise, drinking alcohol and being over-weight are all thought to increase a woman’s risk of developing breast cancer.

**NHS Breast Screening Programme:** A population breast screening programme which was set up in the late 1980’s. All women between the ages of **50-70** are invited for breast screening every 3 years.

**Over-detection**: Some women will be diagnosed and treated for breast cancers that would not have been found without screening and would never have become life-threatening. Doctors cannot always tell whether a breast cancer that is diagnosed will go on to be life-threatening or not, so they offer treatment to all women with a breast cancer. This means that some women will be offered treatment that they do not need. This is known as over-detection. About **3** in every **200** women screened every **3** years from the age of **50-70** are diagnosed with a breast cancer that would never have become life-threatening.

**Reproductive & hormonal history:** As many breast cancers are known to be hormone related, a woman’s childbearing and menstrual history may influence her breast cancer risk. For example, having children early in life and/or multiple pregnancies are known to decrease a woman’s risk of breast cancer. An early menopause, use of oral contraceptives and/or hormone replacement therapy (HRT) can increase a woman’s risk of developing breast cancer.

**Risk of dying from breast cancer**: No cancer screening programme is 100% effective. Even if a woman has regular screening, there is a risk of missing a breast cancer which could progress to become fatal, or of a cancer that develops between screens.

**Risk-based breast screening:** A woman’s breast screening recommendation would be tailored to her personal risk of breast cancer level (e.g., high, moderate, average and low). This could involve more frequent screening for women assessed to be at high or moderate risk and less screening for those at low risk. Women at low risk may be screened for a shorter period during their lifetime than women with a higher risk. The idea here would be to ensure that more women benefit from breast screening and that less are harmed.

## ***S4:* Supplementary analyses**

Testing for random effects

*S4.1.Mixed conditional logit regression model checking for random effects: screening start-age [cmmixlogit choice start0 end65 interval5 interval7 riskdie odx6 odx10, random(start55 start60) noconstant intpoints(5000) difficult]*

| Attribute | Level | Coefficient | p-value* | 95% CI | |
| --- | --- | --- | --- | --- | --- |
| ASC | | | | | |
| **Start age for screening** | ***Age (years)*** |  |  |  |  |
| **50** |  |  |  |  |  |
| **55** |  | **-0.14** | **0.001** | **-0.23** | **-0.05** |
| **60** |  | **-0.67** | **0.0001** | **-0.77** | **-0.56** |
| End age for screening | *Age (years)* |  |  |  |  |
| 70 |  |  |  |  |  |
| 65 |  | -0.34 | 0.0001 | -0.41 | -0.09 |
| Screening intervals | *Years* |  |  |  |  |
| 3 |  |  |  |  |  |
| 5 |  | 0.004 | 0.92 | -0.08 | 0.08 |
| 7 |  | -0.47 | 0.0001 | -0.59 | -0.36 |
| Risk of dying from breast cancer | *Absolute risk* | -0.16 | 0.001 | -0.20 | -0.12 |
| Risk of over-diagnosis |  |  |  |  |  |
| 10 in 1000 |  |  |  |  |  |
| 6 in 1000 |  | 0.10 | 0.04 | 0.01 | 0.19 |
| 2 in 1000 |  | 0.00 | 0.99 | -0.09 | 0.09 |

Log-likelihood ratio (LR) test vs. fixed parameters: χ^2^(2)=0.00, *p*=1.00

*S4.2.Mixed conditional logit regression model checking for random effects: screening end-age [cmmixlogit choice start0 start55 start60 interval5 interval7 riskdie odx6 odx10, random(end65) noconstant]*

| Attribute | Level | Coefficient | p-value* | 95% CI | |
| --- | --- | --- | --- | --- | --- |
| ASC | | | | | |
| Start age for screening | *Age (years)* |  |  |  |  |
| 50 |  |  |  |  |  |
| 55 |  | -0.14 | 0.001 | -0.23 | -0.05 |
| 60 |  | -0.67 | 0.0001 | -0.77 | -0.56 |
| **End age for screening** | ***Age (years)*** |  |  |  |  |
| **70** |  |  |  |  |  |
| **65** |  | **-0.34** | **0.0001** | **-0.41** | **-0.27** |
| Screening intervals | *Years* |  |  |  |  |
| 3 |  |  |  |  |  |
| 5 |  | 0.004 | 0.92 | -0.08 | 0.08 |
| 7 |  | -0.47 | 0.0001 | -0.59 | -0.36 |
| Risk of dying from breast cancer | *Absolute risk* | -0.16 | 0.001 | -0.20 | -0.12 |
| Risk of over-diagnosis |  |  |  |  |  |
| 10 in 1000 |  |  |  |  |  |
| 6 in 1000 |  | 0.10 | 0.04 | 0.01 | 0.19 |
| 2 in 1000 |  | 0.00 | 0.99 | -0.09 | 0.09 |

LR test vs. fixed parameters: χ^2^(1)=0.00, *p*=0.50

*S4.3.Mixed conditional logit regression model checking for random effects: screening intervals [cmmixlogit choice start0 start55 start60 interval5 interval7 riskdie odx6 odx10, random(interval5, interval7) noconstant]*

| Attribute | Level | Coefficient | p-value* | 95% CI | |
| --- | --- | --- | --- | --- | --- |
| ASC | | | | | |
| Start age for screening | *Age (years)* |  |  |  |  |
| 50 |  |  |  |  |  |
| 55 |  | -0.14 | 0.001 | -0.23 | -0.05 |
| 60 |  | -0.67 | 0.0001 | -0.77 | -0.56 |
| End age for screening | *Age (years)* |  |  |  |  |
| 70 |  |  |  |  |  |
| 65 |  | -0.34 | 0.0001 | -0.41 | -0.27 |
| Screening intervals | *Years* |  |  |  |  |
| 3 |  |  |  |  |  |
| 5 |  | **0.004** | **0.92** | **-0.08** | **0.08** |
| 7 |  | **-0.47** | **0.0001** | **-0.59** | **-0.36** |
| Risk of dying from breast cancer | *Absolute risk* | -0.16 | 0.001 | -0.20 | -0.12 |
| Risk of over-diagnosis |  |  |  |  |  |
| 10 in 1000 |  |  |  |  |  |
| 6 in 1000 |  | 0.10 | 0.04 | 0.01 | 0.19 |
| 2 in 1000 |  | 0.00 | 0.99 | -0.09 | 0.09 |

LR test vs. fixed parameters: χ^2^(2)=0.00, *p*=1.00

*S4.4.Mixed conditional logit regression model checking for random effects: risk of dying from breast* cancer [cmmixlogit choice start0 start55 start60 end65 interval5 interval7 od6 od2, random(riskdie) noconstant]

| Attribute | Level | Coefficient | p-value* | 95% CI | |
| --- | --- | --- | --- | --- | --- |
| ASC | | | | | |
| Start age for screening | *Age (years)* |  |  |  |  |
| 50 |  |  |  |  |  |
| 55 |  | -0.14 | 0.001 | -0.23 | -0.05 |
| 60 |  | -0.67 | 0.0001 | -0.77 | -0.56 |
| End age for screening | *Age (years)* |  |  |  |  |
| 70 |  |  |  |  |  |
| 65 |  | -0.34 | 0.0001 | -0.41 | -0.27 |
| Screening intervals | *Years* |  |  |  |  |
| 3 |  |  |  |  |  |
| 5 |  | 0.004 | 0.92 | -0.08 | 0.08 |
| 7 |  | -0.47 | 0.0001 | -0.59 | -0.36 |
| **Risk of dying from breast cancer** | ***Absolute risk*** | **-0.16** | **0.001** | **-0.20** | **-0.12** |
| Risk of over-diagnosis |  |  |  |  |  |
| 10 in 1000 |  |  |  |  |  |
| 6 in 1000 |  | 0.10 | 0.04 | 0.01 | 0.19 |
| 2 in 1000 |  | 0.00 | 0.99 | -0.09 | 0.09 |

LR test vs. fixed parameters: χ^2^(1)=0.00, *p*=0.50

*S4.5.Mixed conditional logit regression model checking for random effects: risk of overdiagnosis* [cmmixlogit choice start0 start55 start60 end65 interval5 interval7 riskdie, random(od6 od2) noconstant]

| Attribute | Level | Coefficient | p-value* | 95% CI | |
| --- | --- | --- | --- | --- | --- |
| ASC | | | | | |
| Start age for screening | *Age (years)* |  |  |  |  |
| 50 |  |  |  |  |  |
| 55 |  | -0.14 | 0.01 | -0.25 | -0.04 |
| 60 |  | -0.73 | 0.0001 | -0.95 | -0.50 |
| End age for screening | *Age (years)* |  |  |  |  |
| 70 |  |  |  |  |  |
| 65 |  | -0.38 | 0.0001 | -0.52 | -0.24 |
| Screening intervals | *Years* |  |  |  |  |
| 3 |  |  |  |  |  |
| 5 |  | 0.004 | 0.98 | -0.09 | 0.09 |
| 7 |  | -0.54 | 0.0001 | -0.74 | -0.33 |
| Risk of dying from breast cancer | *Absolute risk* | -0.17 | 0.001 | -0.23 | -0.12 |
| **Risk of over-diagnosis** |  |  |  |  |  |
| **10 in 1000** |  |  |  |  |  |
| **6 in 1000** |  | **0.09** | **0.16** | **-0.03** | **0.21** |
| **2 in 1000** |  | **-0.01** | **0.83** | **-0.09** | **0.09** |

LR test vs. fixed parameters: χ^2^(2)=0.68, *p*=0.71

Table S4.6 Simultaneous CLM results for participants stratified by survey completion times: T1 = > 8 mins (n = 381) and T2 = < 8 mins (n=121).


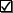
NB: Different attributes do not have the same unit of change so cannot be directly compared with one another. Please see *Table 4* Heat map for RAI of low-risk breast screening scenarios when participants were stratified by age group.

^a^ Coefficient not significantly different from zero; all other coefficients significant at p-value <0.001.

^b^ p-values are from χ^2^ tests that coefficients are equal for the two sub-groups. P-values <0.001 indicate coefficients are significantly different between groups. P-value in the bottom row is for joint test across all coefficients.

| Attribute | Level | Coefficient | p-value* | 95% CI | |
| --- | --- | --- | --- | --- | --- |
| **T1** (< 8 mins**)** | | | | | |
| Start age for screening | *Age (years)* |  |  |  |  |
| 50 |  |  |  |  |  |
| 55 |  | -0.071 | 0.48 | -0.267 | 0.125 |
| 60 |  | -0.7121 | 0.0001 | -0.927 | -0.498 |
| End age for screening | *Age (years)* |  |  |  |  |
| 70 |  |  |  |  |  |
| 65 |  | -0.257 | 0.0001 | -0.402 | -0.112 |
| Screening intervals | *Years* |  |  |  |  |
| 3 |  |  |  |  |  |
| 5 |  | 0.033 | 0.71 | -0.140 | 0 .207 |
| 7 |  |  |  |  |  |
| Risk of dying from breast cancer | *Absolute risk* | -0.128 | 0.001 | -0.204 | -0.053 |
| Risk of over-diagnosis |  |  |  |  |  |
| 10 in 1000 |  |  |  |  |  |
| 6 in 1000 |  | -0.305 | 0.02 | -0.564 | -0.046 |
| 2 in 1000 |  | -0.003 | 0.2 | -0.006 | 0.001 |
| Alternative Specific Constant |  | -2.382 | 0.0001 | -2.758 | -2.006 |
| **T2** (> 8 mins) | | | | | |
| Start age for screening | Age (years) |  |  |  |  |
| 50 |  |  |  |  |  |
| 55 |  | -0.160 | 0.003 | -0.267 | -0.053 |
| 60 |  | -0.641 | 0.001 | -0.762 | -0.521 |
| End age for screening | Age (years) |  |  |  |  |
| 70 |  |  |  |  |  |
| 65 |  | -0.367 | 0.001 | -0.447 | -0.288 |
| Screening intervals | Years |  |  |  |  |
| 3 |  |  |  |  |  |
| 5 |  | -0.002 | 0.96 | -0.100 | 0.096 |
| 7 |  | 0.430 | 0.001 | -0.564 | -0.296 |
| Risk of dying from breast cancer | Absolute risk | -0.171 | 0.001 | -0.213 | -0.128 |
| Risk of over-diagnosis |  |  |  |  |  |
| 10 in 10000 |  |  |  |  |  |
| 6 in 1000 |  | -0.099 | 0.18 | -0.245 | 0.047 |
| 2 in 10000 |  | -0.001 | 0.11 | -0.003 | 0.001 |
| Alternative Specific Constant |  | -2.234 | 0.0001 | -2.434 | -2.036 |
|  |  |  | Χ^2^(9) | P <0.0001 |  |
|  |  | T1 v. T2 | 11.62 | 0.235 |  |


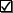
NB: Different attributes do not have the same unit of change so cannot be directly compared with one another. Please see *Table 4* Heat map for RAI of low-risk breast screening scenarios when participants were stratified by age group.

^a^ Coefficient not significantly different from zero; all other coefficients significant at p-value <0.001.

^b^ p-values are from χ^2^ tests that coefficients are equal for the two sub-groups. P-values <0.001 indicate coefficients are significantly different between groups. P-value in the bottom row is for joint test across all coefficients.

## ***S5*. Sub-group analyses**

### *S5.1. Sociodemographic sub-group analysis*

Table S5.1. Simultaneous CLM results for participants stratified by age groups: 40-50, 50-60 & 61-70

| Attribute | Coefficient | Robust std. err | Z | P>\|z\| | 95% CI | |
| --- | --- | --- | --- | --- | --- | --- |
| **40-50 years** (n= 3,984) | | | | | | |
| ASC | -2.43 | .16 | -15.49 | 0.001 | -2.74 | -2.12 |
| Start55 | -.25 | .086 | -3.03 | 0.001 | -.41 | -.09 |
| Start60 | -.87 | .096 | -9.38 | 0.001 | -1.05 | -.69 |
| End65 | -.17 | .066 | -2.74 | 0.006 | -.29 | -.05 |
| Interval 5 | .07 | .08 | 0.97 | 0.334 | -.079 | .22 |
| Interval 7 | -.44 | .108 | -4.21 | 0.001 | -.64 | -.23 |
| Riskdie | -.11 | .03 | -3.44 | 0.001 | -.18 | -.05 |
| Odx6 | -.01 | .08 | -0.12 | 0.905 | -.17 | .15 |
| Odx2 | -.16 | .08 | -1.92 | 0.055 | -.32 | .00 |
|  |  |  |  |  |  |  |
| **51-60 years** (n=4.080) | | | | | | |
| ASC | -2.07 | .15 | -13.78 | 0.001 | -2.36 | -1.77487 |
| Start55 | -.14 | .08 | -1.66 | 0.097 | -.30 | .025 |
| Start60 | -.70 | .09 | -7.37 | 0.001 | -.88 | -.51 |
| End65 | -.32 | .06 | -5.14 | 0.001 | -.44 | -.20 |
| Interval 5 | -.14 | .08 | -1.87 | 0.062 | -.29 | .01 |
| Interval 7 | -.68 | .10 | -6.54 | 0.001 | -.89 | -.48 |
| Riskdie | -.24 | .03 | -7.44 | 0.000 | -.31 | -.18 |
| Odx6 | .16 | .08 | 1.96 | 0.050 | -.00 | .32 |
| Odx2 | .16 | .08 | 1.96 | 0.050 | -.00 | .33 |
| **61-70 years** (n=3,984) | | | | | | |
| ASC | -2.37 | .16 | -14.65 | 0.001 | -2.68 | -2.05 |
| Start55 | -.049 | .08 | -0.58 | 0.561 | -.21 | .12 |
| Start60 | -.47 | .09 | -5.08 | 0.001 | -.65 | -.29 |
| End65 | -.54 | .06 | -8.73 | 0.001 | -.67 | -.42 |
| Interval 5 | .010 | .08 | 1.26 | 0.209 | -.05 | .24 |
| Interval 7 | -.32 | .10 | -3.16 | 0.002 | -.52 | -.12 |
| Riskdie | -.13 | .03 | -3.90 | 0.001 | -.19 | -.06 |
| Odx6 | .14 | .08 | 1.72 | 0.085 | -.02 | .30 |
| Odx2 | -.00 | .08 | -0.05 | 0.964 | -.1670209 | .16 |
|  | Observations N=12,048 |  | *Χ*^2^(9) | *p* |  | |
|  |  | 40-50 vs. 61-70 | 38.03 | 0.0001 *^b^* |  |  |
|  |  | 51-60 vs. 40-50 | 25.60 | 0.0024 |  |  |
|  |  | 61-70 vs. 51-60 | 29.79 | 0.0005 *^b^* |  |  |


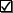
NB: Different attributes do not have the same unit of change so cannot be directly compared with one another. Please see *Table 4* Heat map for RAI of low-risk breast screening scenarios when participants were stratified by age group.

^a^ Coefficient not significantly different from zero; all other coefficients significant at p-value <0.001.

^b^ p-values are from χ^2^ tests that coefficients are equal for the two sub-groups. P-values <0.001 indicate coefficients are significantly different between groups. P-value in bottom row is for joint test across all coefficients.

### *S5.2.* *Attitudes to breast cancer and breast screening*

Table S5.2. **Simultaneous CLM results for participants stratified by breast screening experience: not yet eligible, regular and occasional/non attendees**

| Attribute | Coefficient | Robust std. err | Z | P>\|z\| | 95% CI | |
| --- | --- | --- | --- | --- | --- | --- |
| Not yet eligible (*n*=3,840) | | | | | | |
| ASC | -2.17 | .15 | -14.21 | 0.001 | -2.45 | -1.87 |
| start55 | -.19 | .08 | -2.19 | 0.028 | -.36 | -.02 |
| start60 | -.849 | .09 | -8.99 | 0.001 | -1.03 | -.662 |
| end65 | -.18 | .06 | -2.82 | 0.005 | -.30 | -.05 |
| interval5 | .06 | .08 | 0.74 | 0.458 | -.094 | .21 |
| interval7 | -.44 | .11 | -4.16 | 0.001 | -.65 | -.23 |
| riskdie | -.13 | .03 | -3.88 | 0.001 | -.20 | -.06 |
| od6 | .02 | .08 | 0.28 | 0.781 | -.14 | .19 |
| od2 | -.15 | .09 | -1.76 | 0.078 | -.32 | .02 |
| Regular (*n*=6,744) | | | | | | |
| ASC | -2.74 | .13 | -20.37 | 0.001 | -3.00 | -2.47 |
| Start55 | -.114 | .06 | -1.74 | 0.081 | -.24 | .013 |
| Start60 | -.59 | .07 | -8.07 | 0.001 | -.73 | -.45 |
| End65 | -.47 | .05 | -9.79 | 0.001 | -.56 | -.37 |
| Interval 5 | -.055579 | .06 | -0.95 | 0.341 | -.17 | .06 |
| Interval 7 | -.55 | .08 | -6.86 | 0.001 | -.70 | -.39 |
| Riskdie | -.17 | .03 | -6.83 | 0.001 | -.22 | -.12 |
| Odx6 | .15 | .06 | 2.46 | 0.014 | .03 | .28 |
| Odx2 | .09 | .06 | 1.35 | 0.176 | -.04 | .21 |
| Occasional/never (*n*=1,464) | | | | | | |
| ASC | -1.23 | .22 | -5.50 | 0.001 | -1.66 | -.79 |
| Start55 | -.18 | .14 | -1.32 | 0.186 | -.45 | .09 |
| Start60 | -.61 | .15 | -4.02 | 0.001 | -.90 | -.31 |
| End65 | -.19 | .10 | -1.87 | 0.061 | -.40 | .01 |
| Interval 5 | .15 | .13 | 1.21 | 0.225 | -.10 | .40 |
| Interval 7 | -.23 | .17 | -1.38 | 0.168 | -.55 | .10 |
| Riskdie | -.19 | .05 | -3.46 | 0.001 | -.29 | -.08 |
| Odx6 | .059 | .13 | 0.37 | 0.708 | -.21 | .31 |
| Odx2 | .029 | .14 | 0.15 | 0.880 | -.26 | .30 |
|  | Observations N 12,048 |  | *Χ*^2^( ) | *p* |  | |
|  |  | Regular vs not yet eligible | 47.50 | 0.0001 *^b^* |  |  |
|  |  | Regular vs non_occasional | 64.23 | 0.0001 *^b^* |  |  |
|  |  | Not yet eligible vs non-occasional | 18.48 | 0.03 |  |  |

NB: Different attributes do not have the same unit of change so cannot be directly compared with one another. Please see Table 4 Heat map for RAI of low-risk breast screening scenarios when participants were stratified by breast screening experience.

^a^ Coefficient not significantly different from zero; all other coefficients significant at p-value <0.001.

^b^ p-values are from χ^2^ tests that coefficients are equal for the two sub-groups. P-values <0.001 indicate coefficients are significantly different between groups. P-value in bottom row is for joint test across all coefficients.

Table S5.3. **Simultaneous CLM results for participants stratified by breast cancer worry: high, moderate and low**

| Attribute | Coefficient | Robust std. err | Z | P>\|z\| | 95% CI | |
| --- | --- | --- | --- | --- | --- | --- |
| Low worry (*n*=2,880) |  |  |  |  |  |  |
| ASC | -1.39 | .17 | -8.38 | 0.001 | -1.71 | -1.06 |
| Start55 | -.11 | .10 | -1.13 | 0.260^a^ | -.30 | .08 |
| Start60 | -.49 | .11 | -4.51 | 0.001 | -.71 | -.28 |
| End65 | -.24 | .07 | -3.32 | 0.001 | -.38 | -.10 |
| Interval5 | .14 | .09 | 1.56 | 0.118 ^a^ | -.04 | .32 |
| Interval7 | -.11 | .12 | -0.92 | 0.360 ^a^ | -.34 | .12 |
| Riskdie | -.16 | .04 | -4.28 | 0.001 | -.24 | -.09 |
| Odx2 | .13 | .10 | 1.37 | 0.171 ^a^ | -.06 | .36 |
| Odx6 | .36 | .10 | 3.56 | 0.001 | .166 | .556 |
| Moderate worry (*n*=5,808) |  |  |  |  |  |  |
| ASC | -2.63 | .14 | -18.46 | 0.001 | -2.91 | -2.35 |
| Start55 | -.13 | .07 | -1.53 | 0.126 ^a^ | -.24 | .03 |
| Start60 | -.713 | .08 | -9.11 | 0.001 | -.86 | -.56 |
| End65 | -.45 | .058 | -8.60 | 0.001 | -.55 | -.34 |
| Interval5 | .065 | .068 | 0.98 | 0.325 ^a^ | -.06 | .19 |
| Interval7 | -.52 | .09 | -5.93 | 0.001 | -.69 | -.34 |
| Riskdie | -.18 | .03 | -6.54 | 0.001 | -.23 | -.13 |
| Odx2 | -.03 | .07 | -0.30 | 0.761 ^a^ | -.16 | .11 |
| Odx6 | .13 | .07 | 1.85 | 0.065 ^a^ | -.01 | .26 |
| High worry (*n*=3,360) |  |  |  |  |  |  |
| ASC | -2.61 | .17 | -15.39 | 0.001 | -2.94 | -2.28 |
| Start55 | -.261 | .09 | -2.86 | 0.004 | -.44 | -.082 |
| Start60 | -.75 | .10 | -7.40 | 0.001 | -.95 | -.55 |
| End65 | -.25 | .07 | -3.78 | 0.001 | -.39 | -.129 |
| Interval5 | -.19 | .08 | -2.34 | 0.019 | -.35 | -.03 |
| Interval7 | -.69 | .11 | -6.05 | 0.001 | -.91 | -.47 |
| Riskdie | -.13 | .04 | -3.60 | 0.001 | -.20 | -.06 |
| Odx2 | -.23 | .09 | -2.54 | 0.011 | -.41 | -.05 |
| Odx6 | .03 | .09 | 0.31 | 0.755 ^a^ | -.15 | .20 |
| Observations N=12,048 |  |  | *Χ*^2^( 9) | *p* |  | |
|  |  | High vs. low | 45.81 | 0.0001 *^b^* |  |  |
|  |  | Moderate vs high | 2764 | 0.001 *^b^* |  |  |
|  |  | Moderate vs. Low | 53.14 | 0.0001 *^b^* |  |  |

|  |  |  |  |  |  | |
| --- | --- | --- | --- | --- | --- | --- |
|  | | | | | | |
|  |  |  |  |  |  |  |
|  |  |  |  |  |  |  |
|  |  |  |  |  |  |  |
|  |  |  |  |  |  |  |
|  |  |  |  |  |  |  |
| ASC |  |  |  |  |  |  |
|  | | | | | | |
|  |  |  |  |  |  |  |
|  |  |  |  |  |  |  |
|  |  |  |  |  |  |  |
|  |  |  |  |  |  |  |
|  |  |  |  |  |  |  |
| ASC |  |  |  |  |  |  |
|  | | | | | | |
|  |  |  |  |  |  |  |
|  |  |  |  |  |  |  |
|  |  |  |  |  |  |  |
|  |  |  |  |  |  |  |
|  |  |  |  |  |  |  |
| ASC |  |  |  |  |  |  |
|  |  |  |  |  |  | |
|  |  |  |  |  |  |  |
|  |  |  |  |  |  |  |
|  |  |  |  |  |  |  |

NB: Different attributes do not have the same unit of change so cannot be directly compared with one another. Please see *Table 4* Heat map for RAI of low-risk breast screening scenarios when participants were stratified by intensity and frequency of breast cancer worry.

^a^ Coefficient not significantly different from zero; all other coefficients significant at p-value <0.001.

^b^ p-values are from χ^2^ tests that coefficients are equal for the two sub-groups. P-values <0.001 indicate coefficients are significantly different between groups. P-value in bottom row is for joint test across all coefficients.

Table S5.4. **Simultaneous CLM results for participants stratified by personal experience of friends/family with BC: None, distant and close**

| Attribute | Coefficient | Robust std. err | Z | P>\|z\| | 95% CI | |
| --- | --- | --- | --- | --- | --- | --- |
| None (n=3,312) | | | | | | |
| ASC | -1.77 | .16 | -11.37 | 0.001 | -2.07 | -1.46 |
| Start55 | -.07 | .09 | -0.79 | 0.428 ^a^ | -.25 | .11 |
| Start60 | -.53 | .10 | -5.38 | 0.001 | -.73 | -.34 |
| End65 | -.25 | .07 | -3.72 | 0.001 | -.38 | -.12 |
| Interval 5 | -.04 | .08 | -0.53 | 0.597 ^a^ | -.20 | .12 |
| Interval 7 | -.53 | .11 | -4.69 | 0.001 | -.75 | -.31 |
| Riskdie | -.11 | .04 | -3.10 | 0.002 | -.18 | -.04 |
| Odx6 | .14 | .09 | 1.64 | 0.100 ^a^ | -.03 | .32 |
| Odx2 | .03 | .09 | 0.36 | 0.716 ^a^ | -.15 | .21 |
| Distant (n=7,272) | | | | | | |
| ASC | .03 | .09 | 0.36 | 0.716 ^a^ | -.15 | .21 |
| Start55 | -.18 | .06 | -3.00 | 0.003 | -.31 | -.06 |
| Start60 | -.72 | .07 | -10.44 | 0.001 | -.86 | -.59 |
| End65 | -.39 | .05 | -8.58 | 0.001 | -.48 | -.30 |
| Interval 5 | .01 | .06 | 0.15 | 0.882 ^a^ | -.10 | .12 |
| Interval 7 | -.47 | .08 | -6.18 | 0.001 | -.62 | -.32 |
| Riskdie | -.18 | .02 | -7.24 | 0.001 | -.22 | -.13 |
| Odx6 | .08 | .06 | 1.33 | 0.183 ^a^ | -.04 | .2 |
| Odx2 | .00 | .06 | 0.05 | 0.956 ^a^ | -.12 | .12 |
| Close (n=1,464) | | | | | | |
| ASC | -2.66 | .29 | -9.22 | 0.001 | -3.22 | -2.09 |
| Start55 | -.07 | .14 | -0.48 | 0.628 ^a^ | -.33 | .20 |
| Start60 | -.72 | .16 | -4.47 | 0.001 | -1.03 | -.403 |
| End65 | -.30 | .10 | -2.96 | 0.003 | -.50 | -.10 |
| Interval 5 | .09 | .13 | 0.70 | 0.486 ^a^ | -.16 | .33 |
| Interval 7 | -.41 | .18 | -2.30 | 0.022 | -.77 | -.06 |
| Riskdie | -.20 | .06 | -3.64 | 0.001 | -.31 | -.09 |
| Odx6 | .10 | .13 | 0.71 | 0.475 ^a^ | -.17 | .36 |
| Odx2 | -.08 | .14 | -0.56 | 0.575 ^a^ | -.35 | .19 |
|  | Observations N 12,048 |  | Χ^2^( ) | p |  | |
|  |  | None vs distant | 36.20 | 0.0001 ^b^ |  |  |
|  |  | None vs close | 20.74 | 0.014 |  |  |
|  |  | Distant vs close | 3.86 | 0.92 |  |  |

NB: Different attributes do not have the same unit of change so cannot be directly compared with one another. Please see *Table 4* Heat map for RAI of low-risk breast screening scenarios when participants were stratified by number of personal experiences of family members/friends with a breast cancer diagnosis.

^a^ Coefficient not significantly different from zero; all other coefficients significant at p-value <0.001.

^b^ p-values are from χ^2^ tests that coefficients are equal for the two sub-groups. P-values <0.001 indicate coefficients are significantly different between groups. P-value in bottom row is for joint test across all coefficients.

Table S5.5. **Simultaneous CLM results for participants stratified by prior awareness of overdiagnosis: Yes/No/not sure.**

| Attribute | Coefficient | Robust std. err | Z | P>\|z\| | 95% CI | |
| --- | --- | --- | --- | --- | --- | --- |
| Prior awareness (n=4,776) | | | | | | |
| ASC | -2.70 | .15 | -18.50 | 0.001 | -2.98 | -2.41 |
| Start55 | -.17 | .08 | -2.24 | 0.025 | -.32 | -.02 |
| Start60 | -.68 | .09 | -7.92 | 0.001 | -.85 | -.51 |
| End65 | -.42 | .06 | -7.45 | 0.001 | -.53 | -.31 |
| Interval 5 | -.09 | .07 | -1.38 | 0.167 ^a^ | -.23 | .04 |
| Interval 7 | -.77 | .10 | -7.86 | 0.001 | -.96 | -.58 |
| Riskdie | -.10 | .03 | -3.30 | 0.001 | -.16 | -.04 |
| Odx6 | .03 | .07 | 0.36 | 0.721 ^a^ | -.12 | .17 |
| Odx2 | -.10 | .08 | -1.28 | 0.200 ^a^ | -.25 | .05 |
| No prior awareness (n=6,168) | | | | | | |
| ASC | -1.93 | .13 | -15.44 | 0.001 | -2.18 | -1.69 |
| Start55 | -.10 | .07 | -1.54 | 0.124 ^a^ | -.23 | .028 |
| Start60 | -.64 | .077 | -8.49 | 0.001 | -.78 | -.49 |
| End65 | -.23 | .05 | -5.65 | 0.001 | -.38 | -.18 |
| Interval 5 | .10 | .06 | 1.66 | 0.096 ^a^ | -.02 | .22 |
| Interval 7 | -.22 | .08 | -2.76 | 0.006 | -.38 | -.06 |
| Riskdie | -.22 | .03 | -8.12 | 0.001 | -.27 | -.16 |
| Odx6 | .21 | .07 | 3.21 | 0.001 | .08 | .34 |
| Odx2 | .14 | .07 | 2.03 | 0.043 | .00 | .27 |
| Not sure (n=1,104) | | | | | | |
| ASC | -2.46 | .29 | -8.61 | 0.001 | -3.02 | -1.90 |
| Start55 | -.29 | .16 | -1.73 | 0.083 ^a^ | -.61 | .04 |
| Start60 | -.87 | .18 | -4.78 | 0.001 | -1.22 | -.51 |
| End65 | -.37 | .12 | -3.02 | 0.003 | -.62 | -.13 |
| Interval 5 | -.08 | .15 | -0.54 | 0.590 ^a^ | -.37 | .21 |
| Interval 7 | -.63 | .20 | -3.14 | 0.002 | -1.03 | -.24 |
| Riskdie | -.13 | .06 | -2.09 | 0.037 | -.26 | -.01 |
| Odx6 | -.20 | .16 | -1.22 | 0.221 ^a^ | -.52 | .12 |
| Odx2 | -.33 | .16 | -2.05 | 0.041 | -.64 | -.01 |
|  | Observations N 12,048 |  | Χ^2^( ) | p |  | |
|  |  | Prior vs no awareness | 42.86 | 0.0001 ^b^ |  |  |
|  |  | Prior vs. unsure awareness | 7.80 | 0.55 |  |  |
|  |  | No awareness vs. unsure | 18.09 | 0.03 |  |  |

NB: Different attributes do not have the same unit of change so cannot be directly compared with one another. Please see *Table 4* Heat map for RAI of low-risk breast screening scenarios when participants were stratified prior awareness of overdiagnosis.

^a^ Coefficient not significantly different from zero; all other coefficients significant at p-value <0.001.

^b^ p-values are from χ^2^ tests that coefficients are equal for the two sub-groups. P-values <0.001 indicate coefficients are significantly different between groups. P-value in bottom row is for joint test across all coefficients.

### *S5.3. DCE survey experience*

Table S5.6 **Simultaneous CLM results for participants stratified by participants’ experience of choosing between breast screening choices.**

| Attribute | Coefficient | Robust std. err | Z | P>\|z\| | 95% CI | |
| --- | --- | --- | --- | --- | --- | --- |
| Easy (n=5,112) | | | | | | |
| ASC | -2.39 | .13 | -17.87 | 0.001 | -2.65 | -2.12 |
| Start55 | -.18 | .07 | -2.47 | 0.014 | -.33 | -.038 |
| Start60 | -.59 | .08 | -7.35 | 0.001 | -.75 | -.43 |
| End65 | -.37 | .05 | -6.68 | 0.001 | -.47 | -.26 |
| Interval 5 | -.00 | .07 | -0.04 | 0.966 ^a^ | -.13 | .13 |
| Interval 7 | -.59 | .09 | -6.56 | 0.001 | -.77 | -.42 |
| Riskdie | -.08 | .03 | -2.98 | 0.003 | -.14 | -.03 |
| Odx6 | .01 | .07 | 0.18 | 0.855 ^a^ | -.13 | .15 |
| Odx2 | -.18 | .07 | -2.52 | 0.012 | -.33 | -.04 |
| Neither easy nor difficult (n=2,376) | | | | | | |
| ASC | -1.64 | .19 | -8.73 | 0.001 | -2.012 | -1.27 |
| Start55 | -.10 | .11 | -0.91 | 0.364 ^a^ | -.31 | .11 |
| Start60 | -.79 | .12 | -6.37 | 0.001 | -1.04 | -.55 |
| End65 | -.27 | .08 | -3.29 | 0.001 | -.43 | -.11 |
| Interval 5 | .22 | .10 | 2.17 | 0.030 | .02 | .42 |
| Interval 7 | -.25 | .13 | -1.89 | 0.058 ^a^ | -.52 | .01 |
| Riskdie | -.21 | .04 | -4.88 | 0.001 | -.29 | -.13 |
| Odx6 | .06 | .11 | 0.57 | 0.567 ^a^ | -.15 | .27 |
| Odx2 | .12 | .11 | 1.07 | 0.284 ^a^ | -.10 | .33 |
| Difficult (n=4,560) | | | | | | |
| ASC | -2.63 | .17 | -15.89 | 0.001 | -2.95 | -2.30 |
| Start55 | -.14 | .08 | -1.75 | 0.081 ^a^ | -.29 | .017 |
| Start60 | -.71 | .09 | -8.01 | 0.001 | -.88 | -.54 |
| End65 | -.36 | .06 | -6.32 | 0.001 | -.48 | -.25 |
| Interval 5 | -.08 | .07 | -1.10 | 0.271 ^a^ | -.22 | .06 |
| Interval 7 | -.46 | .10 | -4.72 | 0.001 | -.65 | -.27 |
| Riskdie | -.23 | .03 | -7.28 | 0.001 | -.29 | -.17 |
| Odx6 | .21 | .08 | 2.75 | 0.006 | .06 | .36 |
| Odx2 | .16 | .08 | 1.97 | 0.048 | .00 | .31 |
|  | Observations N 12,048 |  | Χ^2^( ) | p |  | |
|  |  | Easy vs. neither easy nor difficult | 29.70 | 0.0005 ^b^ |  |  |
|  |  | Neither easy nor difficult vs. difficult | 31.32 | 0.0003 ^b^ |  |  |
|  |  | Easy vs difficult | 44.74 | 0.0001 ^b^ |  |  |

NB: Different attributes do not have the same unit of change so cannot be directly compared with one another. Please see *Table 4* Heat map for RAI of low-risk breast screening scenarios when participants were stratified by ease of choosing between alternatives.

^a^ Coefficient not significantly different from zero; all other coefficients significant at p-value <0.001.

^b^ p-values are from χ^2^ tests that coefficients are equal for the two sub-groups. P-values <0.001 indicate coefficients are significantly different between groups. P-value in bottom row is for joint test across all coefficients.

## ***S6****.* *Table S6.1.* Ranked order of the mean predicted probabilities of participant’s selecting all 32 low-risk screening scenarios.

|  |  | | | | |  | | | | |  | |  | |
| --- | --- | --- | --- | --- | --- | --- | --- | --- | --- | --- | --- | --- | --- | --- |
|  |  |  |  |  |  |  |  |  |  |  |  |  |  |  |
|  |  |  |  |  |  |  |  |  |  |  |  |  |  |  |
|  |  |  |  |  |  |  |  |  |  |  |  |  |  |  |
|  |  |  |  |  |  |  |  |  |  |  |  |  |  |  |
|  |  |  |  |  |  |  |  |  |  |  |  |  |  |  |
|  |  |  |  |  |  |  |  |  |  |  |  |  |  |  |
|  |  |  |  |  |  |  |  |  |  |  |  |  |  |  |
|  |  |  |  |  |  |  |  |  |  |  |  |  |  |  |
|  |  |  |  |  |  |  |  |  |  |  |  |  |  |  |
|  |  |  |  |  |  |  |  |  |  |  |  |  |  |  |
|  |  |  |  |  |  |  |  |  |  |  |  |  |  |  |
|  |  |  |  |  |  |  |  |  |  |  |  |  |  |  |
|  |  |  |  |  |  |  |  |  |  |  |  |  |  |  |
|  |  |  |  |  |  |  |  |  |  |  |  |  |  |  |
|  |  |  |  |  |  |  |  |  |  |  |  |  |  |  |
|  |  |  |  |  |  |  |  |  |  |  |  |  |  |  |
|  |  |  |  |  |  |  |  |  |  |  |  |  |  |  |
|  |  |  |  |  |  |  |  |  |  |  |  |  |  |  |
|  |  |  |  |  |  |  |  |  |  |  |  |  |  |  |
|  |  |  |  |  |  |  |  |  |  |  |  |  |  |  |
|  |  |  |  |  |  |  |  |  |  |  |  |  |  |  |
|  |  |  |  |  |  |  |  |  |  |  |  |  |  |  |
|  |  |  |  |  |  |  |  |  |  |  |  |  |  |  |
|  |  |  |  |  |  |  |  |  |  |  |  |  |  |  |
|  |  |  |  |  |  |  |  |  |  |  |  |  |  |  |
|  |  |  |  |  |  |  |  |  |  |  |  |  |  |  |
|  |  |  |  |  |  |  |  |  |  |  |  |  |  |  |
|  |  |  |  |  |  |  |  |  |  |  |  |  |  |  |
|  |  |  |  |  |  |  |  |  |  |  |  |  |  |  |
|  |  |  |  |  |  |  |  |  |  |  |  |  |  |  |
|  |  |  |  |  |  |  |  |  |  |  |  |  |  |  |
|  |  |  |  |  |  |  |  |  |  |  |  |  |  |  |
|  |  |  |  |  |  |  |  |  |  |  |  |  |  |  |
|  |  |  |  |  |  |  |  |  |  |  |  |  |  |  |

|  | **Choice-sets** | | | | | | | | | | | | **CLM coefficients** | | | | | | | | | | | |  |  |  | **Mean predicted**  **probabilities** | |
| --- | --- | --- | --- | --- | --- | --- | --- | --- | --- | --- | --- | --- | --- | --- | --- | --- | --- | --- | --- | --- | --- | --- | --- | --- | --- | --- | --- | --- | --- |
|  | start | | | end | | interval | | | die | odx | | | start | | | end | | interval | | | die | odx | | | ASC | XB | Exp |  |  |
|  | 50 | 55 | 60 | 65 | 70 | 3 | 5 | 7 |  | 10 | 6 | 2 | 50 | 55 | 60 | 65 | 70 | 3 | 5 | 7 |  | 10 | 6 | 2 |  |  |  | **Low** risk | **BSP** |
| BSP | 1 | 0 | 0 | 0 | 1 | 1 | 0 | 0 | 24 | 1 | 0 | 0 | 0 | -0.14 | -0.67 | -0.34 | 0 | 0 | 0.01 | -0.47 | -0.16 | 0 | 0.10 | 0.01 | -2.28 | -6.11 | 0.0022 | 0.50 | 0.50 |
| 6b | 1 | 0 | 0 | 0 | 1 | 1 | 0 | 0 | 24 | 0 | 1 | 0 | 0 | -0.14 | -0.67 | -0.34 | 0 | 0 | 0.01 | -0.47 | 0.16 | 0 | 0.10 | 0.01 | -2.28 | -6.01 | 0.0024 | 0.52 | 0.48 |
| 13a | 0 | 1 | 0 | 0 | 1 | 0 | 1 | 0 | 25 | 0 | 1 | 0 | 0 | -0.14 | -0.67 | -0.34 | 0 | 0 | 0.01 | -0.47 | 0.16 | 0 | 0.10 | 0.01 | -2.28 | -6.31 | 0.0018 | 0.45 | 0.55 |
| 2b | 0 | 1 | 0 | 0 | 1 | 1 | 0 | 0 | 25 | 0 | 0 | 1 | 0 | -0.14 | -0.67 | -0.34 | 0 | 0 | 0.01 | -0.47 | 0.16 | 0 | 0.10 | 0.01 | -2.28 | -6.41 | 0.0016 | 0.43 | 0.57 |
| 8b | 1 | 0 | 0 | 0 | 1 | 0 | 1 | 0 | 26 | 0 | 0 | 1 | 0 | -0.14 | -0.67 | -0.34 | 0 | 0 | 0.01 | -0.47 | 0.16 | 0 | 0.10 | 0.01 | -2.28 | -6.43 | 0.0016 | 0.42 | 0.58 |
| 5b | 1 | 0 | 0 | 1 | 0 | 0 | 1 | 0 | 24 | 0 | 0 | 1 | 0 | -0.14 | -0.67 | -0.34 | 0 | 0 | 0.01 | -0.47 | 0.16 | 0 | 0.10 | 0.01 | -2.28 | -6.45 | 0.0016 | 0.42 | 0.58 |
| 13b | 1 | 0 | 0 | 1 | 0 | 1 | 0 | 0 | 24 | 1 | 0 | 0 | 0 | -0.14 | -0.67 | -0.34 | 0 | 0 | 0.01 | -0.47 | 0.16 | 0 | 0.10 | 0.01 | -2.28 | -6.45 | 0.0016 | 0.42 | 0.58 |
| 4b | 0 | 1 | 0 | 0 | 1 | 0 | 1 | 0 | 26 | 0 | 1 | 0 | 0 | -0.14 | -0.67 | -0.34 | 0 | 0 | 0.01 | -0.47 | 0.16 | 0 | 0.10 | 0.01 | -2.28 | -6.47 | 0.0016 | 0.41 | 0.59 |
| 11b | 1 | 0 | 0 | 1 | 0 | 0 | 1 | 0 | 25 | 0 | 1 | 0 | 0 | -0.14 | -0.67 | -0.34 | 0 | 0 | 0.01 | -0.47 | 0.16 | 0 | 0.10 | 0.01 | -2.28 | -6.51 | 0.0015 | 0.40 | 0.60 |
| 10b | 1 | 0 | 0 | 0 | 1 | 1 | 0 | 0 | 27 | 0 | 0 | 1 | 0 | -0.14 | -0.67 | -0.34 | 0 | 0 | 0.01 | -0.47 | 0.16 | 0 | 0.10 | 0.01 | -2.28 | -6.59 | 0.0014 | 0.38 | 0.62 |
| 2a | 1 | 0 | 0 | 1 | 0 | 0 | 1 | 0 | 26 | 0 | 1 | 0 | 0 | -0.14 | -0.67 | -0.34 | 0 | 0 | 0.01 | -0.47 | 0.16 | 0 | 0.10 | 0.01 | -2.28 | -6.67 | 0.0013 | 0.36 | 0.64 |
| 11a | 0 | 1 | 0 | 0 | 1 | 0 | 0 | 1 | 24 | 0 | 0 | 1 | 0 | -0.14 | -0.67 | -0.34 | 0 | 0 | 0.01 | -0.47 | 0.16 | 0 | 0.10 | 0.01 | -2.28 | -6.73 | 0.0012 | 0.35 | 0.65 |
| 1a | 0 | 1 | 0 | 0 | 1 | 0 | 0 | 1 | 24 | 1 | 0 | 0 | 0 | -0.14 | -0.67 | -0.34 | 0 | 0 | 0.01 | -0.47 | 0.16 | 0 | 0.10 | 0.01 | -2.28 | -6.73 | 0.0012 | 0.35 | 0.65 |
| 9b | 1 | 0 | 0 | 0 | 1 | 0 | 0 | 1 | 25 | 1 | 0 | 0 | 0 | -0.14 | -0.67 | -0.34 | 0 | 0 | 0.01 | -0.47 | 0.16 | 0 | 0.10 | 0.01 | -2.28 | -6.74 | 0.0012 | 0.35 | 0.65 |
| 16a | 0 | 1 | 0 | 1 | 0 | 1 | 0 | 0 | 25 | 0 | 0 | 1 | 0 | -0.14 | -0.67 | -0.34 | 0 | 0 | 0.01 | -0.47 | 0.16 | 0 | 0.10 | 0.01 | -2.28 | -6.75 | 0.0012 | 0.34 | 0.66 |
| 7a | 0 | 0 | 1 | 0 | 1 | 0 | 1 | 0 | 24 | 0 | 0 | 0 | 0 | -0.14 | -0.67 | -0.34 | 0 | 0 | 0.01 | -0.47 | 0.16 | 0 | 0.10 | 0.01 | -2.28 | -6.77 | 0.0011 | 0.34 | 0.66 |
| 3b | 1 | 0 | 0 | 0 | 1 | 0 | 0 | 1 | 26 | 1 | 0 | 0 | 0 | -0.14 | -0.67 | -0.34 | 0 | 0 | 0.01 | -0.47 | 0.16 | 0 | 0.10 | 0.01 | -2.28 | -6.90 | 0.0010 | 0.31 | 0.69 |
| 14b | 0 | 0 | 1 | 0 | 1 | 0 | 1 | 0 | 25 | 1 | 0 | 0 | 0 | -0.14 | -0.67 | -0.34 | 0 | 0 | 0.01 | -0.47 | 0.16 | 0 | 0.10 | 0.01 | -2.28 | -6.93 | 0.0010 | 0.31 | 0.69 |
| 12b | 0 | 0 | 1 | 0 | 1 | 1 | 0 | 0 | 26 | 0 | 1 | 0 | 0 | -0.14 | -0.67 | -0.34 | 0 | 0 | 0.01 | -0.47 | 0.16 | 0 | 0.10 | 0.01 | -2.28 | -7.00 | 0.0009 | 0.29 | 0.71 |
| 3a | 0 | 0 | 1 | 1 | 0 | 1 | 0 | 0 | 24 | 0 | 1 | 0 | 0 | -0.14 | -0.67 | -0.34 | 0 | 0 | 0.01 | -0.47 | 0.16 | 0 | 0.10 | 0.01 | -2.28 | -7.02 | 0.0009 | 0.29 | 0.71 |
| 9a | 0 | 1 | 0 | 1 | 0 | 0 | 1 | 0 | 27 | 0 | 0 | 1 | 0 | -0.14 | -0.67 | -0.34 | 0 | 0 | 0.01 | -0.47 | 0.16 | 0 | 0.10 | 0.01 | -2.28 | -7.07 | 0.0009 | 0.28 | 0.72 |
| 12a | 0 | 1 | 0 | 1 | 0 | 0 | 1 | 0 | 27 | 1 | 0 | 0 | 0 | -0.14 | -0.67 | -0.34 | 0 | 0 | 0.01 | -0.47 | 0.16 | 0 | 0.10 | 0.01 | -2.28 | -7.07 | 0.0009 | 0.28 | 0.72 |
| 7b | 0 | 1 | 0 | 1 | 0 | 1 | 0 | 0 | 27 | 0 | 0 | 1 | 0 | -0.14 | -0.67 | -0.34 | 0 | 0 | 0.01 | -0.47 | 0.16 | 0 | 0.10 | 0.01 | -2.28 | -7.07 | 0.0008 | 0.28 | 0.72 |
| 4a | 1 | 0 | 0 | 1 | 0 | 0 | 0 | 1 | 25 | 0 | 0 | 1 | 0 | -0.14 | -0.67 | -0.34 | 0 | 0 | 0.01 | -0.47 | 0.16 | 0 | 0.10 | 0.01 | -2.28 | -7.09 | 0.0008 | 0.27 | 0.73 |
| 5a | 0 | 0 | 1 | 0 | 1 | 1 | 0 | 0 | 26 | 1 | 0 | 0 | 0 | -0.14 | -0.67 | -0.34 | 0 | 0 | 0.01 | -0.47 | 0.16 | 0 | 0.10 | 0.01 | -2.28 | -7.10 | 0.0008 | 0.27 | 0.73 |
| 10a | 0 | 0 | 1 | 1 | 0 | 0 | 1 | 0 | 24 | 1 | 0 | 0 | 0 | -0.14 | -0.67 | -0.34 | 0 | 0 | 0.01 | -0.47 | 0.16 | 0 | 0.10 | 0.01 | -2.28 | -7.12 | 0.0008 | 0.27 | 0.73 |
| 15b | 0 | 1 | 0 | 1 | 0 | 0 | 0 | 1 | 25 | 0 | 1 | **0** | 0 | -0.14 | -0.67 | -0.34 | 0 | 0 | 0.01 | -0.47 | 0.16 | 0 | 0.10 | 0.01 | -2.28 | -7.13 | 0.0008 | 0.27 | 0.73 |
| 15a | 0 | 0 | 1 | 0 | 1 | 0 | 1 | 0 | 27 | 0 | 0 | 1 | 0 | -0.14 | -0.67 | -0.34 | 0 | 0 | 0.01 | -0.47 | 0.16 | 0 | 0.10 | 0.01 | -2.28 | -7.25 | 0.0007 | 0.24 | 0.76 |
|  | **Choice-sets** | | | | | | | | | | | | **CLM coefficients** | | | | | | | | | | | | |  | | **Mean predicted**  **probabilities** | |
|  | start | | | end | | interval | | | die | odx | | | start | | | end | | interval | | | die | odx | | |  | XB | Exp |  |  |
|  | 50 | 55 | 60 | 65 | 70 | 3 | 5 | 7 |  | 10 | 6 | 2 | 50 | 55 | 60 | 65 | 70 | 3 | 5 | 7 |  | 10 | 6 | 2 | ASC |  |  |  |  |
| 14a | 0 | 1 | 0 | 1 | 0 | 0 | 0 | 1 | 26 | 0 | 1 | 0 | 0 | -0.14 | -0.67 | -0.34 | 0 | 0 | 0.01 | -0.47 | 0.16 | 0 | 0.10 | 0.01 | -2.28 | -7.29 | 0.0007 | 0.24 | 0.76 |
| 6a | 0 | 0 | 1 | 1 | 0 | 0 | 1 | 0 | 26 | 0 | 0 | 1 | 0 | -0.14 | -0.67 | -0.34 | 0 | 0 | 0.01 | -0.47 | 0.16 | 0 | 0.10 | 0.01 | -2.28 | -7.43 | 0.0006 | 0.21 | 0.79 |
| 1b | 0 | 0 | 1 | 1 | 0 | 1 | 0 | 0 | 26 | 0 | 0 | 1 | 0 | -0.14 | -0.67 | -0.34 | 0 | 0 | 0.01 | -0.47 | 0.16 | 0 | 0.10 | 0.01 | -2.28 | -7.44 | 0.0006 | 0.21 | 0.79 |
| 8a | 0 | 0 | 1 | 1 | 0 | 1 | 0 | 0 | 27 | 0 | 1 | 0 | 0 | -0.14 | -0.67 | -0.34 | 0 | 0 | 0.01 | -0.47 | 0.16 | 0 | 0.10 | 0.01 | -2.28 | -7.50 | 0.0006 | 0.20 | 0.80 |
| 16b | 0 | 0 | 1 | 0 | 1 | 0 | 0 | 1 | 27 | 0 | 1 | 0 | 0 | -0.14 | -0.67 | -0.34 | 0 | 0 | 0.01 | -0.47 | 0.16 | 0 | 0.10 | 0.01 | -2.28 | -7.63 | 0.0005 | 0.18 | 0.82 |
|  |  |  |  |  |  |  |  |  |  |  |  |  |  |  |  |  |  |  |  |  |  |  |  |  |  |  |  |  |  |

Key: Breast Screening Programme (BSP); risk of dying (die); risk of overdiagnosis (odx)

## **S7.** **Checklist for conjoint analysis applications in healthcare (Bridges et al., 2011).**

| 1. Was a well-defined research question stated and is conjoint analysis an appropriate method for answering it? | Yes: The study aimed to quantify women’s preferences for low-risk screening scenarios. Breast screening can be considered a preference sensitive healthcare intervention as there may be equipoise involving making trade-offs in line with individual preferences. |
| --- | --- |
| 1.1 Were a well-defined research question and a testable hypothesis articulated? | Yes: The DCE aimed to quantify women’s preferences for a range of low-risk scenarios. It was hypothesised that an earlier end age, later start age, longer screening intervals and increased risk of dying from breast cancer would have a negative impact on perceived utility of low-risk screening scenarios. There was no a priori hypothesis set for overdiagnosis as this is poorly understood by the lay public. |
| 1.2 Was the study perspective described, and was the study placed in a particular decision-making or policy context? | Yes: Women’s screening values and preferences may inform the implementation of a low-risk screening pathway as part of the incorporation of risk-stratification within the NHSBSP. |
| 1.3 What is the rationale for using conjoint analysis to answer the research question? | Yes: A DCE was considered the most appropriate method to quantify women’s preferences for multiple low-risk screening scenarios defined by relevant attributes and their varied levels. |
| 2. Was the choice of attributes and levels supported by evidence? | Yes: Attributes and their levels were informed by a simulated life-table model of RSBS (Pashayan et al., 2018), and ongoing RSBS trials/cohort studies. |
| 2.1 Was attribute identification supported by evidence (literature reviews, focus groups, or other scientific methods)? | Yes: Attributes and their levels were informed by prior research, current literature, and ongoing RSBS trials/cohort studies. |
| 2.2 Was attribute selection justified and consistent with theory? | Yes: Attributes and their levels were informed by prior research, current literature, and ongoing RSBS trials/cohort studies. |
| 2.3 Was level selection for each attribute justified by the evidence and consistent with the study perspective and hypothesis? | Yes: Attributes and their levels were informed by prior research, current literature, and ongoing RSBS trials/cohort studies. |
| 3. Was the construction of tasks appropriate? | Yes: PPI consultation and a pilot study were conducted to check usability and comprehensibility of DCE tasks. |
| 3.1 Was the number of attributes in each conjoint task justified (that is, full or partial profile)? | Yes: The scenarios were defined by 5 attributes which was considered a manageable number in terms of mitigating cognitive burden. Age range of screening eligibility and screening intervals are features of the screening programme , whilst risk of dying from breast cancer and overdiagnosis represented features of the screening test. Nevertheless, there are other features of breast screening that could be used by future studies. |
| 3.2 Was the number of profiles in each conjoint task justified? | Yes: PPI consultation and a pilot study were conducted to check usability and comprehensibility of DCE tasks. |
| 3.3 Was (should) an opt-out or a status-quo alternative (be) included? | Yes: As breast screening attendance is not mandatory in the UK |
| 4. Was the choice of experimental design justified and evaluated? | Yes: We liaised with a health economist, Stephen Morris, who has experience of designing choice experiments. |
| 4.1 Was the choice of experimental design justified? Were alternative experimental designs considered? | No |
| 4.2 Were the properties of the experimental design evaluated? | Yes: Orthogonality and level-balance checks were carried out. |
| 4.3 Was the number of conjoint tasks included in the data-collection instrument appropriate? | Yes: 8 choice tasks/participant is considered a manageable number (Lancsar and Louviere, 2012). |
| 5. Were preferences elicited appropriately, given the research question? | Yes: Elicited preferences were in line with study aims to quantify women’s preferences for low-risk screening pathways where there may be equipoise between the benefits and harms. |
| 5.1 Was there sufficient motivation and explanation of conjoint tasks? | Yes: PPI consultation and a pilot study were conducted to check usability and comprehensibility of DCE tasks. A risk communication expert was also consulted. |
| 5.2 Was an appropriate elicitation format (that is, rating, ranking, or choice) used? Did (should) the elicitation format allow for indifference? | Yes: One choice/participant with an option to choose ‘no screening’. |
| 5.3 In addition to preference elicitation, did the conjoint tasks include other qualifying questions (for example, strength of preference, confidence in response, and other methods)? | No |
| 6. Was the data collection instrument designed appropriately? | Yes (S2) |
| 6.1 Was appropriate respondent information collected (such as sociodemographic, attitudinal, health history or status, and treatment experience)? | Yes: See Study procedure and materials (S3). |
| 6.2 Were the attributes and levels defined, and was any contextual information provided? | Yes: Survey information materials and hyperlinks to further information about the attributes and their levels were embedded within choice questions (S3). |
| 6.3 Was the level of burden of the data-collection instrument appropriate? Were respondents encouraged and motivated? | Yes: 8 choice-sets is manageable (Lancsar & Louviere, 2012) and the survey included non-DCE measures to assess participants understanding and overall survey experience. |
| 7. Was the data-collection plan appropriate? | Yes: An online research panel was used (Savanta) |
| 7.1 Was the sampling strategy justified (for example, sample size, stratification, and recruitment)? | Yes: See: Quotas for SES and age were specified for the recruitment of 500 participants. This sample size provided power for exploratory sub-group analyses. |
| 7.2 Was the mode of administration justified and appropriate (for example, face-to-face, pen-and-paper, web-based)? | Yes: A web-based was considered to be a practical option given time and financial constraints. |
| 7.3 Were ethical considerations addressed (for example, recruitment, information and/or consent, compensation)? | Yes: The study secured ethical approval from the Bio-medical Panel, King’s College London, (Ref: LRS-21/22-25904). |
| 8. Were statistical analyses and model estimations appropriate? | Yes: Conditional logit regression models and post-estimation methods including computation of RAI and mean predicted probabilities. |
| 8.1 Were respondent characteristics examined and tested? | Yes: Extensive sub-group analyses were conducted. |
| 8.2 Was the quality of the responses examined (for example, rationality, validity, reliability)? | Yes: Visual inspections and sensitivity analyses were conducted for speeder and satisficers. |
| 8.3 Was model estimation conducted appropriately? Were issues of clustering and subgroups handled appropriately? | Yes |
| 9. Were the results and conclusions valid? | Yes. See Results and Discussion |
| 9.1 Did study results reflect testable hypotheses and account for statistical uncertainty? | Yes. See Statistical analyses and Results |
| 9.2 Were study conclusions supported by the evidence and compared with existing findings in the literature? | Yes. See Discussion |
| 9.3 Were study limitations and generalizability adequately discussed? | Yes: See Strengths and limitations |
| 10. Was the study presentation clear, concise, and complete? | Yes |
| 10.1 Was study importance and research context adequately motivated? | Yes: See DCE survey templates (S3) |
| 10.2 Were the study data-collection instrument and methods described? | Yes: The development of the DCE survey and data collection procedure are described in the Methods section. and procedure |
| 10.3 Were the study implications clearly stated and understandable to a wide audience? | Yes: The implications of the study results are relevant to researchers of cancer screening behaviour and health policy makers. Although public preferences will be key to whether and/or how risk-stratification is implemented within the NHSBSP, the current study has yet to be reported to a lay audience. |
